# Integrated Space-Time Dataset Reveals High Diversity and Distinct Community Structure of Ciliates in Mesopelagic Waters of the Northern South China Sea

**DOI:** 10.3389/fmicb.2019.02178

**Published:** 2019-09-24

**Authors:** Ping Sun, Liying Huang, Dapeng Xu, Alan Warren, Bangqin Huang, Ying Wang, Lei Wang, Wupeng Xiao, Jie Kong

**Affiliations:** ^1^Key Laboratory of the Ministry of Education for Coastal and Wetland Ecosystem, College of the Environment and Ecology, Xiamen University, Xiamen, China; ^2^Fujian Provincial Key Laboratory for Coastal Ecology and Environmental Studies, Xiamen University, Xiamen, China; ^3^State Key Laboratory of Marine Environmental Science, Institute of Marine Microbes and Ecospheres, College of Ocean and Earth Sciences, Xiamen University, Xiamen, China; ^4^Department of Life Sciences, Natural History Museum, London, United Kingdom

**Keywords:** ciliates, diversity, community structure, assembly mechanism, high throughput cDNA sequencing, quantitative protargol stain, mesopelagic zone

## Abstract

Little is known about diversity distribution and community structure of ciliates in mesopelagic waters, especially how they are related to spatial and temporal changes. Here, an integrative approach, combining high-throughput cDNA sequencing and quantitative protargol stain, was used to analyze ciliate communities collected temporally along a transect from coastal to oceanic regions at depths ranging from the surface to 1000 m. The mesopelagic zone exhibited comparable alpha diversity to surface water which was consistent over temporal variation, with high diversity occurring at the interface with the euphotic zone. Comparison with the northeastern and the western Pacific Ocean revealed consistency of this vertical distribution of ciliates across oceanic basins. Mesopelagic ciliates harbored distinct community structure without significant seasonal differences, with the vertical variations driven largely by members of the classes Spirotrichea and Oligohymenophorea. Operational taxonomic units (OTUs) affiliated with Scuticociliatia, Astomatida and Apostomatida, members of which are known to be bacterivorous and/or commensal/parasitic species, were more abundant in mesopelagic waters than above, implying they are an important component of food webs in the mesopelagic zone. A combination of depth, geographic distance and environment shaped the ciliate communities, with depth being the most influential factor. Phylogenetic null modeling analysis further indicated that 57.1 and 33.3% of mesopelagic community variation was governed by dispersal limitation and heterogeneous selection, respectively, probably due to the marked biochemical and physical gradients down the water column. This suggests that ciliate community structure in the mesopelagic zone is mainly controlled by stochastic processes. Collectively, this study reports mesopelagic ciliates exhibited high diversity and distinct community structure across spatiotemporal scales and informs the processes mediating ciliate assembly in the mesopelagic zone. These should be fully considered in future studies to build a more comprehensive understanding of mesopelagic microbial assemblages.

## Introduction

The mesopelagic realm is a vast, dimly lit region that spans the globe between about 200–1,000 m beneath the ocean’s surface. It is also the zone where 90% of the organic carbon exported from epipelagic waters is remineralized (Robinson et al., 2010). By their grazing on particulate organic matter such as bacteria and other microorganisms, ciliates act as active regulators of biogeochemical processes and nutrient cycling and thus play key roles in mesopelagic ecosystem function (Tanaka and Rassoulzadegan, 2002; Gowing et al., 2003). Therefore, knowledge of ciliate diversity distribution, community structure and assembly mechanisms are needed in order to improve understanding of the microbial ecology in the mesopelagic zone.

Despite limited studies, contrasting findings have been reported in terms of ciliate diversity distribution (Wickham et al., 2011; Grattepanche et al., 2016). For example, both microscopy- and 18S rRNA gene clone library-based studies showed that ciliate abundance and species richness decrease with the increasing water depth, the low abundance of prokaryotes as a food resource in the waters below the euphotic zone being cited as the most likely cause (Tanaka and Rassoulzadegan, 2002; Countway et al., 2010; Wickham et al., 2011). By contrast, a study of two major lineages of planktonic ciliates off the coast of New England employing high-throughput sequencing (HTS) of the hyper variable V2 region of the rRNA gene revealed that alpha-diversity increased with increasing depth, the highest diversity occurring below the photic zone (50–800 m) (Grattepanche et al., 2016). In order to resolve this discrepancy, studies that employ a combination of both morphology- and molecular-based methods to analyze ciliate diversity down the water column over spatial and temporal scales are required.

Compared with the photic zone, studies of mesopelagic ciliates are limited and mainly focus on their abundance and biomass (Gowing et al., 2003; Hu et al., 2016; Zhao et al., 2017; Dolan et al., 2019). Addressing the assembly mechanisms impacting microbial communities, which is important to strengthen our understanding of the ecological processes mediating community assembly of microorganisms, has recently received more attention (Stegen et al., 2013; Zhou and Ning, 2017; Logares et al., 2018). The relative importance of deterministic vs. stochastic processes depends on spatial and temporal scales, species traits and the local environment (Dolan et al., 2007; Doherty et al., 2010) which, to the best of our knowledge, have never been evaluated for mesopelagic ciliates.

The South China Sea (SCS) is one of the largest marginal seas in the western Pacific Ocean, with an area of about 3.3 million km^2^ and a depth ranging from the shallowest coastal fringe to a maximum of more than 5000 m (Liang et al., 2008). The northeastern SCS is connected to the western Pacific Ocean by the Luzon Strait, which has a sill depth of about 1900 m. The SCS is located within the range of the monsoon regime and is affected by the southwest monsoon in summer and the northeast monsoon in winter (Morton and Blackmore, 2001).

In this study, a combination of morphology-based quantitative protargol stain (QPS) and 18S rRNA gene transcript-based HTS approaches were employed to investigate the diversity and community structure of ciliates along a coast-to-oceanic transect in the northern region of the SSC. Samples were collected at various depths from the surface to the mesopelagic zone (1000 m deep) in spring and summer of the years 2013 and 2014, respectively. Employing morphological or molecular approaches, previous studies have revealed a distinct vertical distribution of alpha diversity, i.e., diversity increases/decreases with depth (Countway et al., 2010; Wickham et al., 2011; Grattepanche et al., 2016). The first question explored here is whether or not the diversity of ciliates down the water column, revealed by a combination of morphological and molecular approaches, is consistent with either of the previous findings. Mesopelagic ciliates occupy distinct ecological niches and play differential ecological functions compared to members of the photic zone (Gowing et al., 2003; Zhao et al., 2017). Therefore, the second question explored here is the membership of the mesopelagic ciliate assemblage differs from that of overlying communities. Thirdly, we investigate whether mesopelagic ciliates show temporal patterns of distribution and the ecological processes that shape ciliate assembly in mesopelagic zone.

## Materials and Methods

### Sampling and Environmental Factors Analyses

Samples were collected from five sites separated by 35.7–50.0 km in a transect from 33 km to 207 km off the coast of Guangdong Province (China) during spring (April 24–26th, 2013; April 13–16th, 2014) and summer (June 28–30th, 2013; July 27–29th, 2014) on board the R/V Yanping II (Supplementary Figure S1). The transect is located between the Taiwan Strait and the SCS. The Taiwan Strait is characterized by a complex bottom topography and is located mostly on the continental shelf with a mean depth of about 60 m. An abrupt depth change is present between the Taiwan Strait and the SCS deep basin, with deepest water depth over 1000 m (Hu et al., 2011). The transect crosses coastal (water depth < 50 m), slope break (water depth < 200 m) and oceanic (water depth up to 1197 m) areas in the northern SCS (Hu et al., 2011). Sites C1 to C5 are in shallow systems, with depth around 30–60 m (Supplementary Table S1). Only surface waters (at a depth of 3 m) were collected. At sites C7 and C9, for which deepest depth are around 130 and 1300 m, respectively, samples were collected at discrete depths (Supplementary Table S1). During the study period, a total of 41 samples were collected, with 18 samples from the surface, 16 samples from the euphotic zone (surface layer not included) and 7 samples from the mesopelagic zone (Supplementary Table S1). All samples were collected using a conductivity temperature depth (CTD) profiler equipped with 12-l Niskin bottles (SBE 917, United States). The bulk sample was prefiltered through 200 μm nylon mesh (Sefar Nitex) to remove mesozooplanktonic organisms. A subsample (2 L) of the pre-filtered water was passed through a 3 μm pore polycarbonate membrane (Millipore, United States), which was then immersed in preservative buffer (RNAlater, Qiagen) and stored at −20°C until extraction. For the QPS, subsamples (500 ml) of water were collected in spring and summer of the year 2014 from the bulk waters. The samples were fixed immediately with freshly prepared Bouin’s preservative according to Montagnes and Lynn (1993).

Temperature, salinity, and depth of all water samples were measured using a conductivity–temperature–depth (CTD) profiler. For chlorophyll *a* analysis, 200 ml seawater was passed through a GF/F filter, which was immediately stored at −80°C for later extraction (Huang et al., 2010). Acetone was used to extract chlorophyll *a*, the concentration of which was measured by Trilogy fluorometer (Turner Designs, United States). For bacterial abundance analysis, which was only conducted for the samples collected in 2014, 1.8 ml seawater sample was pre-filtered onto a 20 μm pore-size membrane and then mixed with ice-cold glutaraldehyde (1% final concentration). Samples were stored at −80°C and were later measured by a flow cytometry (Beckman Coulter, Epics Altra II) (Jiao et al., 2013). Nutrient analysis (ammonia, nitrite, nitrate, dissolved inorganic nitrogen and phosphate) followed Hu et al. (2008).

### Quantitative Protargol Stain, Nucleic Acid Extraction and PCR

The 22 Bouin’s-fixed samples were filtered through a 25 mm diameter 0.8 μm pore-size cellulose nitrate membrane (Sartorius, Lot no: 11404-25), bleached and stained using the QPS (Montagnes and Lynn, 1993). All isolates were observed using an Olympus CX31 microscope under bright field to reveal taxonomically informative features such as the infraciliature and nuclear apparatus. Ciliate identification followed Kofoid and Campbell (1929, 1939) and Song et al. (2009). The abundance of each species was recorded.

In total, 41 samples collected during spring and summer of 2013 and 2014 were processed for HTS of the 18S rRNA gene transcript. For RNA extraction, a RNeasy Mini Kit (Qiagen, United States) was used following the removal of RNAlater buffer (Stoeck et al., 2010). The extracted RNA was treated with the RNase-free DNase set (Qiagen, United States) to remove remaining DNA. Reverse transcription was performed using QuantiTect Reverse Transcription Kit (Qiagen, United States). The PCR amplification of ciliate partial 18S rRNA gene transcript was performed using ciliate-specific primers (Dopheide et al., 2008). Primers covering the hypervariable V4 region of the 18S rRNA gene transcript were then used in a second PCR following Stoeck et al. (2010). Q5 Hot Start High-Fidelity DNA Polymerase (Cat. #M0493 L, New England Biolabs, United States) was used for fragment amplification. PCR conditions used for the first PCR follows Dopheide et al. (2008) with a few modifications. Details are as follow: an initial incubation for 5 min at 98°C and then 25 cycles of 60 s at 98°C (denaturing), 30 s at 55°C (annealing), and 90 s at 72°C (extension), followed by a final extension step of 7 min at 72°C. PCR conditions used for the second PCR follow Stoeck et al. (2010) with a few modifications. Details are as follows: an initial activation step at 98°C for 5min, followed by 25 three-step cycles consisting of 98°C for 30 s, 57°C for 45 s, and 72°C for 1 min; and a final 7 min extension at 72°C. Each sample was amplified in triplicate, pooled, and purified using the Wizard^®^ SV Gel and PCR Clean-Up System (Promega, United States). All purified fragments were paired-end sequenced using Illumina MiSeq platform by the sequencing company Meiji (Shanghai, China). All Illumina Miseq raw sequence data were deposited in NCBI Sequence Read Archive (accession code PRJNA517357).

### Sequence Data Analyses

Cleaning of raw sequence data was performed with Trimmomatic (Bolger et al., 2014) and Flash (Magoč and Salzberg, 2011), including quality checking and filtering, demultiplexing, and assembly of data. The criteria were as follows: (i) low quality reads that had an average quality score lower than 20 and were shorter than 50 bp were removed; (ii) reads that contained ambiguous characters, a mismatch in barcode and/or two or more mismatches in primer, were discarded; (iii) reads with an overlapping region less than 10 bp, or with a mismatch ratio more than 0.2, were removed. The resulting clean reads were further processed using USEARCH v10 (Edgar, 2010) and were clustered at 97% sequence similarity level. *De novo*-based chimera removal was implemented in the clustering process. UCHIME implemented in USEARCH v10 was used to perform analysis of reference-based chimera removal (Edgar et al., 2011). Representative sequences were then taxonomically assigned against the Protist Ribosomal Reference database v4.7.2 (PR2) by UBLAST (Guillou et al., 2013). Non-ciliate OTUs were manually removed and the generated ciliate-only OTU table was ready for further analyses. Prior to subsequent statistical analyses, the OTU table was rarefied to the same sequencing depth of 7,597 reads per sample by randomly resampling. For samples collected in spring and summer of the year 2014, the OTU table was normalized to 7,532 reads per sample for further analyses.

In order to further explore whether the diversity distribution pattern seen in our study is a general phenomenon, we downloaded HTS data generated from two pilot studies carried out in neighboring oceanic basins, i.e., western Pacific Ocean (Zhao et al., 2017) and eastern North Pacific (Hu et al., 2016), respectively. Both investigations amplified the same hypervariable region, i.e., V4, as that of the present study, derived from HTS of both DNA and cDNA, thus enabling direct comparisons to be made. Sequence data from the eastern North Pacific were downloaded from the NCBI Sequence Read Archive under accession number SRP070577 (Hu et al., 2016). Sequence data from the western Pacific were downloaded from the NCBI Sequence Read Archive under accession number SRP109014 (Zhao et al., 2017). For the eastern North Pacific, 25 cDNA samples were extracted which encompassed 70,250 ciliate sequences. For the western Pacific, 23 DNA and 6 cDNA samples were extracted which included totals of 739,909 and 202,116 ciliate sequences, respectively. Details of extract locations and depths are given in Supplementary Table S2. All data processing was the same as the one dealing with the data from the present study.

### Statistical Analyses

Alpha-diversity estimators were calculated using SPADE (Chao and Shen, 2003) and diversity comparisons on spatial and temporal scales were depicted by boxplots. Sampling sites were defined as nearshore when the water depth was less than 50 m and offshore when the water depth was more than 50 m. To investigate differences between samples, Bray-Curtis distances were calculated and analyzed by Non-Metric Multidimensional Scaling (nMDS) in R using the ‘vegan’ package^1^. For samples collected in spring and summer of the year 2014, the groupings of samples were identified using PCoA on both morphological and V4 amplicon datasets with the ‘vegan’ package in R. Global and pairwise differences among groupings of samples were further tested by analysis of similarity (ANOSIM), permutational multivariate analysis of variance (Adonis) and multiple response permutation procedures (MRPP) within R and PRIMER 6 (Clarke and Gorley, 2009). SIMPER analysis was employed to identify those OTUs responsible for the differences observed in the composition across depth strata in PRIMER 6. Indicator species analysis was performed to identify the specific OTUs that characterized each of the ecological layers using the package Indicspecies in R (Dufrene and Legendre, 1997). Only OTUs with indicator values (IV) > 0.3 and *p* < 0.05 were considered good indicators. The relationships between communities and environmental factors were explored with Mantel tests using the vegan package in R. Partial Mantel tests were used to assess individual effects of depth, geographical distance and environment factors on beta-diversity after controlling for each (Legendre and Legendre, 1998). Quantification of ecological processes, i.e., selection, dispersal and drift, were made according to Stegen et al. (2013) which first uses phylogenetic turnover between communities to determine the influence of selection, then uses OTU turnover to determine the influences of dispersal and drift. Phylogenetic turnover was measured by calculating the weighted β-mean nearest taxon distance (βMNTD), which quantifies the mean phylogenetic distance between the evolutionary closest OTUs in two communities. βMNTD values higher than expected indicate that communities are under heterogeneous selection (Zhou and Ning, 2017). In contrast, βMNTD values which are lower than expected indicate that communities are experiencing homogeneous selection. Null models were constructed using 999 randomizations as in Stegen et al. (2013). Differences between the observed βMNTD and the mean of the null distribution are denoted as β−Nearest Taxon Index (βNTI). βNTI values higher than 2 or lower than −2 indicate the influence of the deterministic process structuring the community, whereas βNTI values between −2 and 2 indicate the influence of stochastic processes (Stegen et al., 2013). The second step is to calculate whether the observed β diversity, based in OTU turnover, could be generated by drift or other processes. The Raup-Crick metric (Chase et al., 2011) using Bray–Curtis dissimilarities, was calculated following Stegen et al. (2013). RCbray compares the measured β diversity against the β diversity that would be obtained if drift was driving community turnover (i.e., under random community assembly). The randomization was run 999 times. Then the βNTI in combination with RCbray was used to quantify the relative influence of major ecological processes governing the ciliate communities (Stegen et al., 2013). βNTI values higher than 2 or lower than −2 indicate the influence of variable selection and homogeneous selection, respectively. If the |βNTI| is <2 but with an RCbray value lower than −0.95 or higher than +0.95, the community assembly is governed by homogenizing dispersal or dispersal limitation, respectively. In addition, a |βNTI| of <2 and a |RCbray| of <0.95 suggest that the community assembly is not dominated by any single process (Stegen et al., 2013).

## Results

### Environmental Parameters

Environmental parameters collected along the transect showed gradients in both dimensions, i.e., horizontally from coastal to oceanic waters and vertically from surface to deep waters. Variations occurring horizontally along the transect were linked mainly to increases in temperature and salinity and a decrease in turbidity from coastal to oceanic waters, whereas variations occurring vertically down the water column were linked to a decrease in temperature and increases in pressure, salinity, and concentrations of nitrate, phosphate and dissolved inorganic nitrogen, from surface to deep waters (Table 1 and Supplementary Figure S1). Environmental factors such as temperature, salinity, pressure, dissolved oxygen, pH, abundances of bacteria and concentration of nutrients co-varied significantly with water depth (Supplementary Table S3), suggesting strong vertical variability in environmental features. In addition, the interface (100–200 m) between the euphotic and mesopelagic zones is highly heterogeneous, and represents a layer of major transitions of several factors, i.e., concentrations of dissolved oxygen, nitrite, nitrate, phosphate and dissolved inorganic nitrogen, temperature, turbidity and pH (Table 1). For ease of description, terms used to describe water layers at different depths are defined here. Water at the depth of 3 m is defined as surface; water between 50 and 100 m is defined as euphotic zone; water between 200 and 1000 m is defined as mesopelagic zone; water between 100 and 200 m is defined as transitional zone, i.e., the layer between the base of the euphotic zone and the top of the mesopelagic zone.

**TABLE 1:** Environmental parameters at sampling sites throughout period of study (mean ± SD).

**Sampling sites**	**C1 3 m**	**C3 3 m**	**C5 3 m**	**C7 3 m**	**C9 3 m**	**C7 50–100 m**	**C9 50–100 m**	**C9 200 m**	**C9 1000 m**
Salinity (‰)	33.21 ± 1.28	32.93 ± 1.52	33.58 ± 0.64	33.77 ± 0.34	33.92 ± 0.35	34.43 ± 0.22	34.37 ± 0.24	34.52 ± 0.01	34.56 ± 0.04
Temperature (°C)	25.06 ± 2.00	26.90 ± 3.29	27.22 ± 2.85	27.32 ± 2.02	27.36 ± 2.16	21.34 ± 3.45	23.54 ± 2.39	15.22 ± 1.17	4.36 ± 0.44
DO (mg/L)	6.83 ± 0.28	6.67 ± 0.61	6.50 ± 0.59	6.23 ± 0.21	6.63 ± 0.28	5.79 ± 0.53	5.70 ± 0.51	3.92 ± 0.65	3.23 ± 0.13
Pressure (db)	3.69 ± 1.16	3.52 ± 1.01	3.52 ± 1.01	3.69 ± 1.16	3.03 ± 0.02	79.62 ± 29.88	72.32 ± 24.94	201.30 ± 0.11	1008.60 ± 0.55
pH	8.46 ± 0.13	8.39 ± 0.17	8.39 ± 0.17	8.47 ± 0.12	8.33 ± 0.08	8.34 ± 0.15	8.27 ± 0.07	8.13 ± 0.06	7.86 ± 0.06
Turbidity (FTU)	1.33 ± 1.26	0.80 ± 0.29	0.61 ± 0.00	0.50 ± 0.10	0.50 ± 0.04	0.58 ± 0.04	0.53 ± 0.08	0.26 ± 0.10	0.61 ± 0.00
Chl *a* (μg/L)	1.51 ± 0.99	0.34 ± 0.20	0.13 ± 0.02	0.13 ± 0.09	0.11 ± 0.06	0.24 ± 0.19	0.35 ± 0.17	0.02 ± 0.04	0.00 ± 0.00
Bacteria (10^4^cells/mL)	136.15 ± 4.95	107.44 ± 29.10	103.24 ± 38.76	91.45 ± 32.76	70.96 ± 11.16	68.23 ± 37.41	54.61 ± 19.62	23.65 ± 2.17	8.70 ± 3.70
DIN (mg/L)	0.016 ± 0.003	0.010 ± 0.010	0.014 ± 0.008	0.024 ± 0.019	0.016 ± 0.012	0.060 ± 0.033	0.047 ± 0.041	0.158 ± 0.019	0.416 ± 0.058
NO_2_ (mg/L)	0.002 ± 0.002	0.002 ± 0.002	0.002 ± 0.002	0.001 ± 0.001	0.001 ± 0.001	0.002 ± 0.001	0.003 ± 0.003	0.001 ± 0.001	0.001 ± 0.001
NO_3_ (mg/L)	0.012 ± 0.006	0.006 ± 0.010	0.008 ± 0.008	0.017 ± 0.014	0.013 ± 0.011	0.055 ± 0.035	0.043 ± 0.041	0.154 ± 0.016	0.409 ± 0.054
NH_4_ (mg/L)	0.002 ± 0.003	0.003 ± 0.006	0.004 ± 0.004	0.006 ± 0.005	0.002 ± 0.003	0.003 ± 0.005	0.002 ± 0.002	0.002 ± 0.003	0.006 ± 0.003
PO_4_ (mg/L)	0.003 ± 0.003	0.002 ± 0.002	0.002 ± 0.002	0.003 ± 0.003	0.005 ± 0.003	0.011 ± 0.006	0.010 ± 0.009	0.028 ± 0.003	0.079 ± 0.007

*DO and pH were not measured for samples collected in the summer of 2013, and bacteria were not enumerated for samples collected in the spring and summer of 2013.*

### Diversity Distribution and Community Structure of Ciliates Revealed by Both Morphology and rRNA Gene Transcript Datasets (Year 2014)

#### Alpha- and Beta-Diversity Distribution Patterns and Their Relations to Spatial and Temporal Variations

**FIGURE 1:**
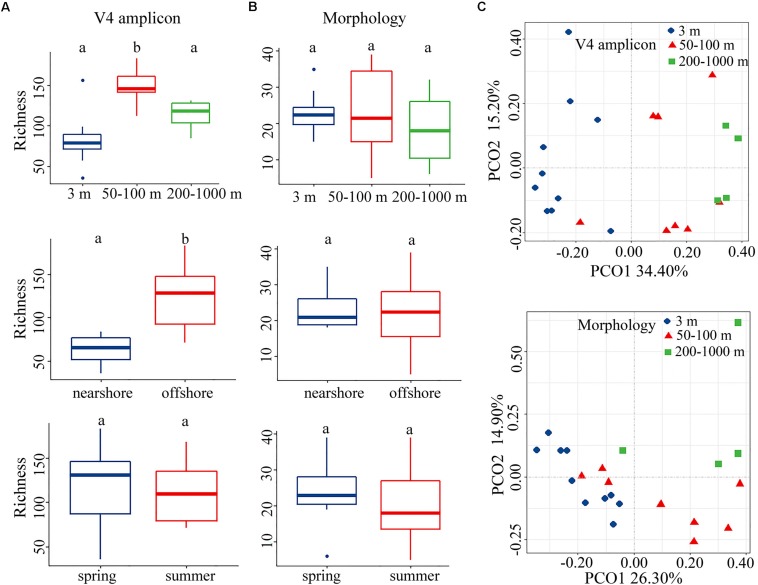
**(A,B)** Comparison of operational taxonomic unit (OTU) or species richness in vertical (upper), horizontal (middle), and seasonal (lower) dimensions revealed by molecular (left) and morphological (right) approaches for the year 2014. Bars without shared letters indicate significant differences at the level of *p* = 0.05. **(C)** Principal coordinate analysis (PCoA) plot for the year 2014 based on Bray–Curtis dissimilarity revealed by the molecular (upper) and morphological (lower) approaches.

We compared distribution patterns, rather than the exact values, of alpha-diversity using two approaches, i.e., morphology and rRNA gene transcripts. In contrast to morphospecies richness, OTU richness had a clear spatial distribution, both horizontally and vertically (Figures 1A,B, upper and middle). When samples were considered by ecological layers (surface, euphotic zone, and mesopelagic zone), there was a marked increase of OTU richness from the surface to the euphotic waters (Figure 1A, upper and Supplementary Table S4). Both approaches revealed that the mesopelagic zone possessed comparable diversity to that of surface layer (Figures 1A,B and Supplementary Table S4), with higher diversity occurring on the top of the mesopelagic zone (Supplementary Figures S2a,b), which was consistent with the result from the pooled data for the years 2013 and 2014 (Supplementary Figure S3a). This vertical distribution pattern of alpha-diversity was consistent over seasonal and annual timescales. In contrast to the clear spatial distribution, differences of alpha-diversity between seasons (spring-summer) were statistically insignificant (Figures 1A,B, lower and Supplementary Table S4). This was consistent with the results from the pooled data for the years 2013 and 2014 which showed that ciliate alpha-diversity had a clear spatial, but no temporal, distribution pattern (Supplementary Figure S3b).

**FIGURE 2:**
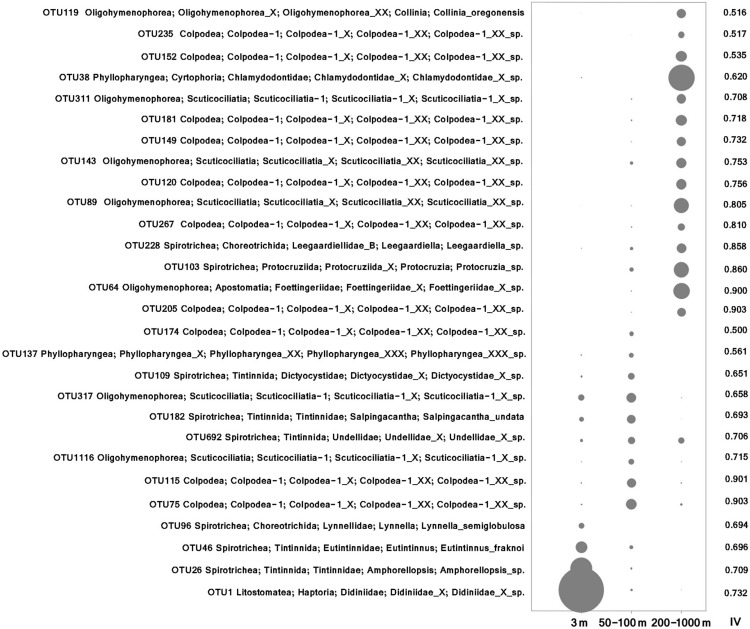
Bubble plot of indicator OTUs in surface (3 m), euphotic (50–100 m) and mesopelagic (200–1000 m) layers. The size of the bubble indicates the average relative abundance of each OTU in each ecological layer. Indicator values (IV) are displayed in the right column.

Both morphological and molecular approaches captured similar vertical distribution patterns of ciliate communities based on PCoA, ANOSIM, Adonis and MRPP analyses, identifying three ciliate assemblages that were mainly grouped by water depth, i.e., the surface, euphotic, and mesopelagic zones groups (Figure 1C and Table 2). SIMPER analysis showed that there are two major contributors to the vertical community rearrangements, i.e., Spirotrichea (which contributed 29.05 and 62.03% to the molecular and morphological datasets, respectively) and Oligohymenophorea (which contributed 27.57 and 21.62% to the molecular and morphological datasets, respectively). Although the relative sequence abundance of spirotricheans declined with depth from surface to euphotic zone in both datasets, it increased from the euphotic to mesopelagic zones in the molecular dataset but decreased in the morphological dataset (Supplementary Table S5). The relative sequence abundance of oligohymenophoreans increased with depth in both datasets (Supplementary Table S5). In addition, we observed a change in indicator species in different ecological layers, e.g., OTUs 1, 26, and 46, which showed a high similarity to an environmental ciliate sequence (KJ763408, coverage 100%, similarity 100%), *Amphorellopsis* sp. (KF130398, coverage 100%, similarity 100%), and *Eutintinnus fraknoi* (JQ408159, coverage 100%, similarity 99%), respectively, made a high contribution to the surface water communities. By contrast, OTUs 38, 64, 103, and 89, which were closely related to an uncultured eukaryote sequence from a high-Arctic fjord, Isfjorden in West Spitsbergen, Norway (KT810411, coverage 98%, similarity 99%), an uncultured deep sea (1500–2500 m) eukaryote sequences from East Pacific Rise, North Pacific (KJ761209, KJ760912, KJ757205, coverage 100%, similarity 100%), an uncultured eukaryote sequences from coastal North Pacific (KJ762665, coverage 100%, similarity 99%) and a Gulf Stream, North Atlantic (KJ760065, coverage 100%, similarity 99%), and an uncultured apostome ciliate (JX417930, coverage 100%, similarity 100%), respectively, dominated the mesopelagic zone (Figure 2). The community structure in both the surface and euphotic zones exhibited seasonal changes (Table 2). The molecular dataset also revealed the presence of a distance-decay pattern in the community composition from coastal to oceanic waters (Table 2). By contrast, although the morphological dataset revealed a significant difference in community composition between spring and summer, it failed to detect a nearshore-offshore distribution pattern (Table 2). Beta-diversity based on the pooled rRNA dataset for the years 2013 and 2014 also captured a clear spatial, but no temporal distribution pattern (Supplementary Table S6).

**TABLE 2:** ANOSIM, Adonis, and MRPP statistical tests of the groupings of molecular- and morphology-based ciliate communities according to depth, region and season.

	**ANOSIM**				**Adonis**				**MRPP**			
			
	**2014_V4**		**2014_Morphology**		**2014_V4**		**2014_Morphology**		**2014_V4**		**2014_Morphology**	
						
	***R***	***P***	***R***	***P***	***R*^2^**	***P***	***R*^2^**	***P***	***A***	***p***	***A***	***p***
Depth (*N* = 22)	0.66	**<0.001**	0.392	**<0.001**	0.375	**0.001**	0.25	**0.001**	0.169	**0.001**	0.088	**0.001**
3 m vs. 50–100 m *N*(3 m) = 10, *N*(50–100 m) = 8	0.6	**<0.001**	0.288	**0.002**	0.284	**0.002**	0.143	**0.006**	0.13	**0.001**	0.047	**0.006**
3 m vs. 200–1000 m *N*(3 m) = 10, *N*(200–1000 m) = 4	0.879	**0.002**	0.66	**0.004**	0.353	**0.002**	0.277	**0.002**	0.157	**0.002**	0.105	**0.002**
50–100 m vs. 200–1000 m *N*(50–100 m) = 8, *N*(200–1000 m) = 4	0.572	**0.008**	0.276	0.072	0.259	**0.008**	0.195	**0.022**	0.096	**0.003**	0.06	**0.021**
Nearshore vs. offshore *N*(nearshore) = 4, N(offshore) = 18	0.353	**0.008**	–0.038	0.531	0.138	**0.003**	0.06	0.153	0.052	**0.005**	0.012	0.103
Season (spring vs. summer) *N*(spring) = 11, *N*(summer) = 11	0.021	0.273	0.126	**0.019**	0.056	0.243	0.084	**0.046**	0.007	0.227	0.02	**0.039**
Spring vs. summer (3 m) *N*(spring) = 5, *N*(summer) = 5	0.188	0.077	0.556	**0.007**	0.178	0.143	0.252	**0.009**	0.031	0.088	0.082	**0.009**
Spring vs. summer (50–100 m) *N*(spring) = 4, *N*(summer) = 4	0.056	0.347	0.135	0.196	0.184	0.129	0.151	0.272	0.041	0.088	0.002	0.313

*Community turnover is based on the Bray–Curtis distance. Numbers in bold indicate statistically significant results.*

Spirotrichea and Oligohymenophorea dominated ciliate communities from the surface down to mesopelagic zone in the morphological dataset in the year 2014 (Supplementary Figure S4a), with the former contributing higher sequence abundance to the communities than that of the latter at the surface and in the euphotic zone whereas in the mesopelagic zone the opposite was true (Supplementary Figure S4a). Together with Phyllopharyngea, these two classes were significant contributors to the ciliate communities across three depth strata, i.e., surface, euphotic and mesopelagic zones, in the molecular dataset in the year 2014 (Supplementary Figure S4b), reflecting the results of the pooled dataset for the years 2013 and 2014 (Supplementary Figure S4b). Spirotricheans, the largest representatives in the ciliate communities, decreased from surface to euphotic zone in both morphological and molecular datasets (Supplementary Figures S4a–c). Spirotrichea remained the dominant class in the mesopelagic zone in the molecular dataset in the year 2014 (Supplementary Figure S4b). By contrast, Oligohymenophorea was the dominant class below the euphotic zone in the morphological dataset in the year 2014 (Supplementary Figure S4a) and in the pooled dataset for years 2013 and 2014 (Supplementary Figure S4c). The relative contributions of Oligohymenophorea and Phyllopharyngea to ciliate communities down the water column showed opposite trends, that of the former increasing with water depth in all datasets (Supplementary Figures S4a–c) whereas that of the latter decreasing with water depth in molecular datasets (Supplementary Figures S4b,c).

#### Determinants of Ciliate Community Dynamics

Depth, geographic distance and environmental factors were generally correlated with ciliate beta-diversity, the only exception being that between geographic distance and community variation in the morphological dataset (Table 3). The partial Mantel test revealed that depth made a greater contribution to community variation, with higher R values of depth (ranging from 0.485 to 0.593) than those of geographic distance (from 0.370 to 0.395) and environment (from 0.004 to 0.414) (Table 3). These results were also reflected in the plots of depth, geographic distance and environmental heterogeneity versus community dissimilarity, all of which had positive slopes, with depth showing the highest correlation (Supplementary Figures S5a–c). Depth and environmental factors explained both molecular- and morphology-based ciliate community variability, with statistically significant relationships occurring more frequently for OTUs than for morphospecies (Table 3). Statistically significant results were revealed for depth after controlling for environmental factors, while all putatively correlated factors showed either a decrease in correlation coefficient or an insignificant effect on morphology-based beta-diversity when depth was controlled (Table 3).

**TABLE 3:** Simple and partial Mantel tests for the correlations between spatial/environmental factors and ciliate communities revealed by the rRNA gene transcript and morphology datasets for the year 2014.

**Mantel test**	**2014_V4**		**2014_morphology**		**Partial Mantel test control for**	**2014_v4**		**2014_Morphology**	
					
	***R***	***P***	***R***	***P***		***R***	***P***	***R***	***P***
Depth	0.585	**< 0.001**	0.41	**< 0.001**	Geo_distance	0.593	**< 0.001**	0.384	**< 0.001**
					Environment	0.485	**< 0.001**	0.296	**< 0.001**
Geo_distance	0.426	**< 0.001**	0.086	0.242	Depth	0.37	**0.001**	0.056	0.3
					Environment	0.395	**< 0.001**	0.01	0.422
Environment	0.380	**< 0.001**	0.241	**0.039**	Depth	0.004	0.434	–0.0001	0.457
					Geo_distance	0.414	**< 0.001**	0.261	**0.029**
Pressure	0.585	**< 0.001**	0.41	**< 0.001**	Depth	–0.147	0.941	–0.19	0.965
Temperature	0.385	**0.006**	0.345	**0.045**	Depth	0.258	**0.014**	0.134	0.161
Salinity	0.357	**0.004**	0.007	0.393	Depth	0.216	**0.041**	–0.18	0.939
Turbidity	0.033	0.349	–0.072	0.646	Depth	0.053	0.31	–0.113	0.769
Chl *a*	0.096	0.203	0.013	0.364	Depth	0.052	0.283	–0.081	0.71
Bacteria	0.537	**< 0.001**	0.426	**0.007**	Depth	0.267	**0.004**	0.124	0.143
DIN	0.410	**0.003**	0.425	**0.015**	Depth	0.104	0.198	0.247	0.051
NO_2_	–0.017	0.524	–0.173	0.963	Depth	0.119	0.097	–0.097	0.851
NO_3_	0.412	**0.003**	0.425	**0.017**	Depth	0.114	0.169	0.23	0.063
NH_4_	–0.04	0.656	0.079	0.192	Depth	–0.048	0.699	0.078	0.176
PO_4_	0.410	**0.001**	0.368	**0.025**	Depth	0.197	**0.048**	0.199	0.072

*Numbers of samples for statistical analyses are 22. Numbers in bold indicate statistically significant results.*

**FIGURE 3:**
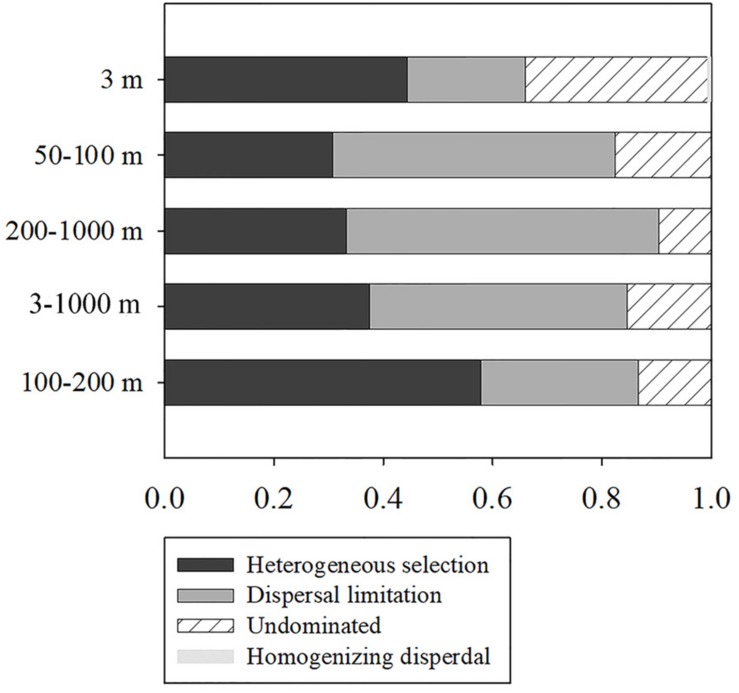
Summary of the relative contributions of the ecological processes that determine community assembly at different depths.

To further assess the contributions of spatial and environmental factors on ciliate community structure, quantification of the ecological processes mediating community assembly was performed with the phylogenetic null model analysis (Figure 3). It was found that dispersal limitation, which generates divergence in community composition due to limited exchange of microbes, was primary for total community and explained 47.2% of community turnover (Figure 3). Although the combined effects of non-selection processes explained more of the variation of the surface community, heterogeneous selection itself, which causes community composition to be dissimilar under variable environmental conditions, explained 44.4% of the variation. For euphotic and mesopelagic zones, dispersal limitation (51.7 and 57.1%, respectively) and heterogeneous selection (30.8 and 33.3%, respectively) were the major contributors to community turnover, while ecological drift which results from population sizes fluctuating due to chance events, contributed 17.5 and 9.5%, respectively (Figure 3). Considering the high ciliate diversity in the transitional layer, the processes shaping community assembly in this layer were also analyzed. In contrast to the other layers, heterogeneous selection contributed 57.8% of community variation, whereas dispersal limitation and ecological drift explained only 28.9 and 13.3% of the variation (Figure 3).

### Ciliate Taxonomy and Abundance Revealed by QPS

**FIGURE 4:**
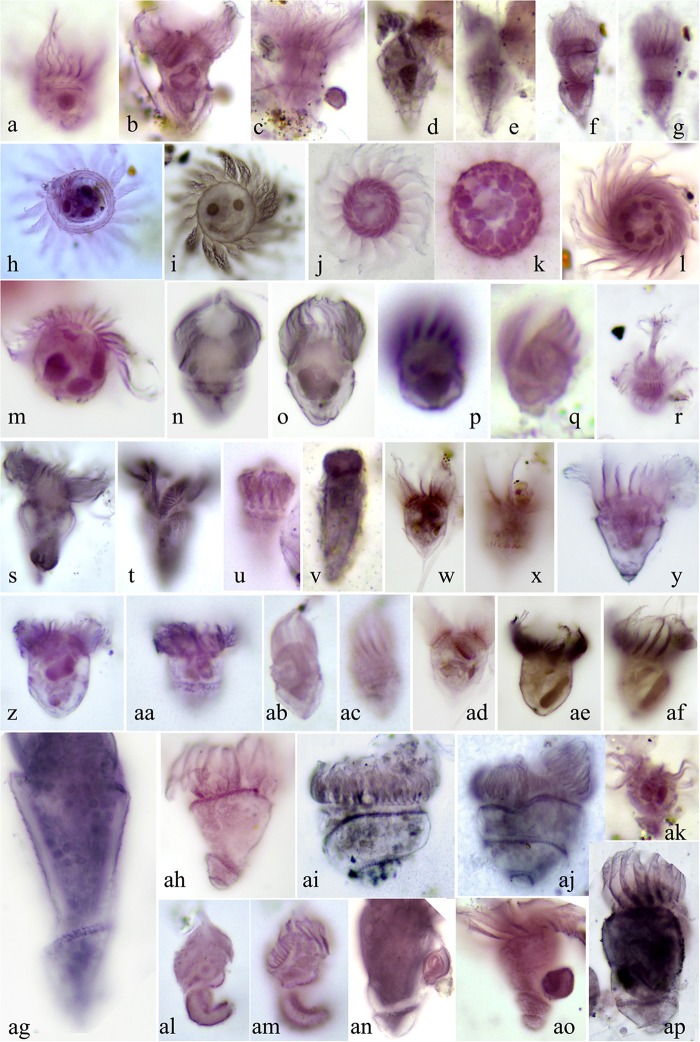
Morphology of representative oligotrichians and aloricate choreotrichians following protargol staining. **(a)** *Leegaardiella ovalis*. **(b,c)** *Strombidium bilobum*. **(d,e)** *Strombidium dalum* sensu Lynn et al. (1988). **(f,g)** *Strombidium emergens*. **(h)** *Leegaardiella* cf. *sol*. **(i)** *Leegaardiella sol*. **(j–l)** *Rimostrombidium multinucleatum*. **(m)** *Lohmanniella* sp2. **(n,o)** *Strombidium* sp3. **(p,q)** *Strombidium pollostomum*. **(r)** *Mesodinium* sp. **(s,t)** *Pseudotontonia cornuta*. **(u,v)** *Strombidinopsis* sp. **(w,x,ad)** *Strombidium styliferum*. **(y)** *Strombidium constrictum*. **(z,aa)** *Strombidium* cf. *inclinatum*. **(ab,ac)** *Strombidium dalum*. **(ae,af)** *Strombidium* sp4. **(ag)** *Loboea strobila* Lohmann, 1908. **(ah)** *Loboea* sp. **(ai,aj)** *Parallelostrombidium kahli*. **(ak)** Tontoniidae sp. **(al,am)** *Paratontonia gracillima*. **(an)** *Spirotontonia turbinata*. **(ao)** *Spirotontonia grandis*. **(ap)** *Spirotontonia* sp.

In total, 79 morphospecies were recovered based on QPS analysis, representing 29 genera, 23 families, 9 orders, 7 subclasses and 5 classes (Supplementary Table S7). Among the seven subclasses, species of Oligotrichia (31.33%), Choreotrichia (28.35%), Scuticociliatia (19.77%) and Hapotoria (16.32%) contributed over 95% of the total abundance. The most abundant subclass, the aloricate Oligotrichia, showed great diversity in terms of morphology (Figure 4). Several isolates appear to be new to science given the combination of their unique infraciliature and minute body size (10–20 μm). These will be reported in detail in a separate taxonomic paper. At order and family ranks, members of Strombidiida and Strombidiidae had the highest abundances, accounting for 31.32% and 30.75% of the total abundance, respectively. *Strombidium* was the most abundant genus among the 29 genera recovered, contributing 30.72% of the total abundance. The three most abundant species were from classes Hapotoria and Spirotrichea, i.e., a species of Didiniidae (8.74%), a species of Tontoniidae (6.48%) and *Strombidium* sp5 (6.42%), collectively contributing 21.64% of the total ciliate abundance.

In spring and summer of 2014, average ciliate abundances in surface waters were 2,120 ind/L and 1,509 ind/L, respectively, as revealed by QPS. In both seasons, the inshore average abundances (on average 2,006 ind/L in spring and 1,366 ind/L in summer) of ciliates in surface waters were lower than those offshore (on average 2,290 ind/L in spring and 1,723 ind/L in summer) (Supplementary Figure S6). Vertically, the maximum abundance of ciliates occurred at 50 m at all offshore sites, with 5,451 ind/L at C7-50m in spring and 3,533 ind/L at C9–50 m in summer (Supplementary Figure S6). The minimum abundances all appeared at 1000 m of C9 in both seasons (Supplementary Figure S6).

As depth was the most influential factor for community variability, the dominant species above and below photic zone were also analyzed. The same three species that dominated species abundance of the total community also dominated the photic zone, i.e., a species of Didiniidae (8.89%), a species of Tontoniidae (6.54%) and *Strombidium* sp5 (6.35%), which collectively contributed 21.78% of the abundance in waters of the 3–100 m depth range. In the aphotic zone, *Strombidium* sp5 (9.25%) and two species of Scuticociliatia (7.09 and 6.89%, respectively) were the three most abundant species, collectively contributing 23.23% of ciliate abundance in waters of the 200–1000 m depth range. Interestingly, *Strombidium* sp5 was found to be one of the three most abundant species in both the photic and the aphotic zones.

The ciliate community also showed seasonal variation (Table 2) so the dominant species in spring and summer were analyzed. The same three species that dominated species abundance of the total community also dominated the spring community, i.e., a species of Didiniidae (9.94%), a species of Tontoniidae (8.14%) and *Strombidium* sp5 (7.20%), which collectively contributed 25.28% of the abundance in spring community. In summer, a species of Didiniidae (6.63%), a species of Scuticociliatia (5.48%) and *Leegaardiella ovalis* (5.34%) were the three most abundant species, collectively contributing 17.45% of ciliate abundance.

### Mesopelagic Ciliates Across Oceanic Basins

**FIGURE 5:**
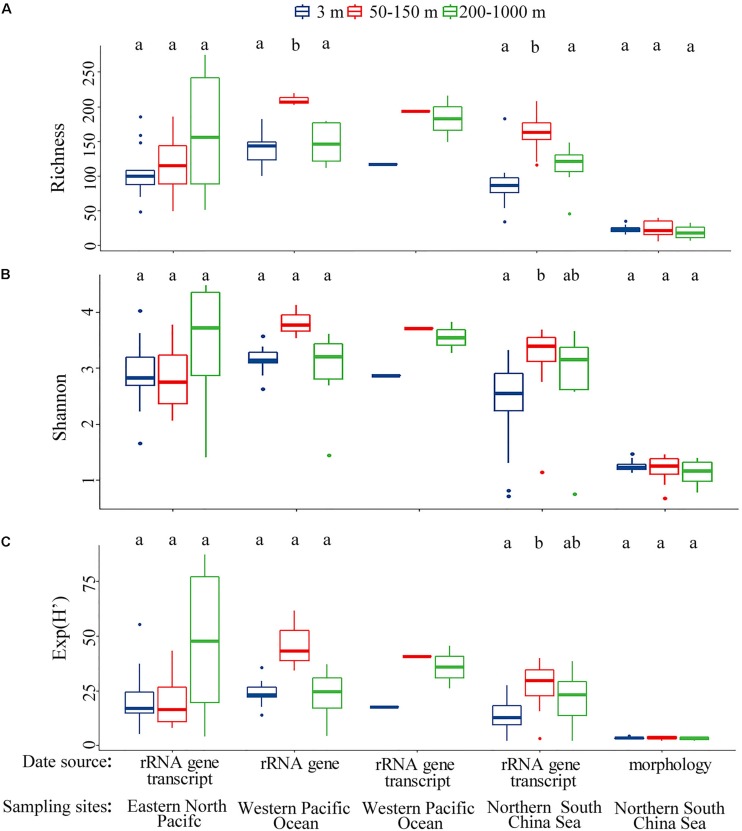
Alpha diversity metrics including **(A)** OTU/species richness, **(B)** Shannon’s *H*’ and **(C)** effective number of species [exp(H’)] in vertical distribution derived from samples collected from the eastern North Pacific, western Pacific Ocean and northern South China Sea based on multiple data sources (i.e., rRNA gene transcript, rRNA gene, and morphology). To evaluate the consistency of the alpha diversity in vertical distribution among the following metrics were computed: **(A)** OTU/species richness (considering observed richness), **(B)** Shannon’s *H*’ (considering both richness and relative abundance), and **(C)** effective number of species [exp(H’)] (considering true diversity, not indices). Bars without shared letters indicate significant differences at the level of *p* = 0.05.

**FIGURE 6:**
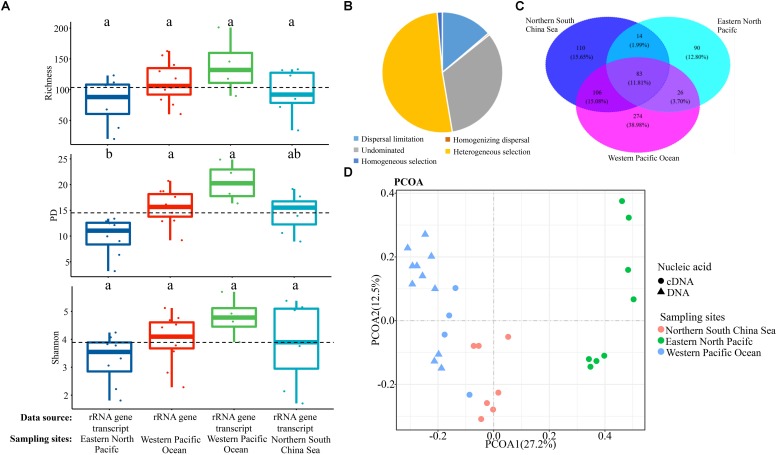
**(A)** OTU richness (upper), phylogenetic diversity (PD, middle), and Shannon (lower) indices and **(B)** community distribution of mesopelagic ciliates derived from samples collected from the eastern **(C,D)** North Pacific, western Pacific Ocean and northern South China Sea based on multiple data sources (i.e., rRNA gene transcript and rRNA gene). Bars without shared letters indicate significant differences at the level of *p* = 0.05.

Both morphological and molecular approaches showed that the mesopelagic ciliates possessed comparable diversity to that of the surface layer in the northern SCS (Figures 1A,B upper). To further test how this diversity distribution related to spatial changes, datasets from the western Pacific Ocean (Zhao et al., 2017) and eastern North Pacific (Hu et al., 2016) were analyzed. These revealed a consistent distribution pattern in each of the three oceanic basins regardless of the type of dataset analyzed, i.e., rRNA gene, rRNA gene transcript or morphology (Figures 5A–C). Generally, alpha diversity indices (richness, phylogenetic diversity and Shannon) of mesopelagic ciliates across oceanic basins showed insignificant differences, except for the eastern North Pacific vs. the western Pacific Ocean regarding phylogenetic diversity (Figure 6A and Supplementary Table S8). About 12.80–38.98% of all OTUs in the mesopelagic dataset were unique to each oceanic region (Figure 6B). Among the total of 703 OTUs, 11.81% were shared by all three basins, whereas OTUs shared by any two basins accounted for 1.99–15.08%, that between the northern SCS and the eastern North Pacific being lowest (Figure 6C). PCoA revealed that mesopelagic samples from different oceanic basins were clearly separated from each other in their community composition (Figure 6D). The analysis of the global R statistics by the ANOSIM demonstrated that differences in mesopelagic ciliate communities between oceanic basins were significantly different. In addition, pairwise comparisons of the mesopelagic ciliate communities clearly indicated that oceanic basins differed from each other, although the difference between the two neighboring basins, i.e., northern SCS and western Pacific Ocean, was lower than other pairwise comparisons (Supplementary Table S9). Phylogenetic null model analysis showed that heterogeneous selection was responsible for 51.26% of compositional variation of mesopelagic ciliate communities across basins, whereas ecological drift and dispersal limitation accounted for 33.10 and 13.79% of community variation, respectively (Figure 6B).

## Discussion

### Diversity Distribution of Ciliates in Mesopelagic Waters Across Spatiotemporal Scales

Although studies on ciliate distribution down the water column, especially below the euphotic zone, are limited, different distribution patterns of alpha-diversity have previously been reported (Tanaka and Rassoulzadegan, 2002; Countway et al., 2010; Wickham et al., 2011; Grattepanche et al., 2016). A microscopy-based study showed low biomass and abundance of ciliates below the euphotic zone in the northwestern Mediterranean Sea (Tanaka and Rassoulzadegan, 2002). Likewise, the ciliate alpha-diversity declined with depth less steeply than biomass and abundance below the euphotic zone in the Amundsen and Bellingshausen Seas (Wickham et al., 2011). Using clone libraries, a lower abundance of ciliate OTUs was found in the waters below than above the euphotic zone in North Pacific Ocean, with alpha-diversity decreasing with increasing depth (Countway et al., 2010). By contrast, using pyrosequencing of rRNA genes, alpha-diversity increased with increasing depth for oligotrichians and choreotrichians, with the highest diversity occurring below the euphotic zone along a 163 km transect off the coast of New England, United States (Grattepanche et al., 2016). In the present study, we observed neither a decrease nor an increase of alpha-diversity in the mesopelagic zone. Rather, the rRNA gene transcript-based data revealed that the mesopelagic zone possessed comparable diversity to that of the surface (Figure 1A and Supplementary Figure S3a). This finding was mirrored by the morphology-based data (Figure 1B). The sharp contrast between consistent patterns shown here and the lack of consistent patterns in previous studies could be due to methodological differences and/or local characteristics of the different oceanic regions. HTS methods have recently been developed which can detect species even if present in low number, thereby avoiding the bias that leads to underestimates the diversity of small, cryptic and low abundance species using morphology-based and classical molecular-based methods (Santoferrara et al., 2014). Previous studies investigating temporal changes in species richness and abundance of mesopelagic tintinnids in the North West Mediterranean Sea (Dolan et al., 2019), and in biomass of mesopelagic ciliates in the Arabian Sea (Gowing et al., 2003) have yielded contrasting findings which likely reflected differences in the two ecosystems examined. The mesopelagic zone is not uniform, but contains a strong gradient of environmental factors, especially at the interface with the euphotic zone (Dehairs et al., 1997; Robinson et al., 2010). These environmental gradients may support a wide range of niches that can be occupied by organisms with different functionality. Furthermore, the steep environmental gradients, both physical and chemical, may prevent strong dominance by any particular species, thus helping to create biodiversity hotspots. This is exemplified by the significantly lower ciliate abundance, but comparable alpha diversity, in the mesopelagic zone compared to the surface (Figure 1B and supplementary Figure S6).

### Ciliate Community Structure and Assembly Mechanisms Across Depth Strata

There are marked biochemical and physical gradients down the water column from the surface to the mesopelagic zone. This provides a structured environment and generates different ecological layers, leading to strong dispersal limitation and environmental selection among layers and within each layer. Different ciliate assemblages are associated with these ecological layers and two classes in particular, Spirotrichea and Oligohymenophorea, contributed significantly to differences among the vertical communities (Supplementary Table S5). In the case of Spirotrichea (mainly Oligotrichia and Choreotrichia), abundance of OTUs affiliated to loricate choreotrichs (tintinnids) was lower in the mesopelagic zone (14.84%) than in layers above (18.72%) whereas oligotrichs together with aloricate choreotrichs showed an opposite trend (82.81% in mesopelagic vs. 77.02% in layers above). This is mirrored by morphological data that showed the relative abundance of loricate choreotrichs was lower in the mesopelagic zone (13.28%) than in layers above (20.23%) whereas the relative abundance of oligotrichs together with aloricate choreotrichs was higher in mesopelagic (86.72%) than in layers above (78.84%). Further analysis showed that loricate and aloricate ciliates accounted for 32.35% and 66.29% of spirotrichean community variations, respectively. Prey was found to be an important beta-diversity driver of tintinnid communities across the shelf off the coast of Rhode Island (United States) and small-sized tintinnids were proposed to consume bacteria in bottom waters of sampling sites (32–120 m) (Santoferrara et al., 2016). Gómez (2007) found that aloricate choreotrichs and oligotrichs were less affected than tintinnids by the reduction of food availability under oligotrophic conditions. Aloricate ciliates, therefore, would be expected to dominate the spirotrichean community in mesopelagic waters given that the food sources, e.g., pico- and nano-phytoplankton, flagellates and bacteria, are generally less accessible than in the layers above (Christaki et al., 1999; Pitta et al., 2001). Tintinnid diversity was found to more closely reflect resource diversity than competitive interactions or predation (Dolan et al., 2002). In the present study, we found that tintinnid diversity in mesopelagic waters was much lower than that of the other layers, indicating a possible low diversity of tintinnid food source in the mesopelagic zone. Among oligohymenophoreans, the relative abundance of OTUs affiliated with Scuticociliatia increased from 65.4% in the euphotic zone to 90.7% in the mesopelagic zone. Furthermore, scuticociliates contributed 62.11% to the differences in oligohymenophorean communities down the water column. Scuticociliates are able to withstand a wide range of salinities and are particularly abundant in habitats with high levels of organic matter, nutrients and bacteria (Urrutxurtu et al., 2003). The higher abundance of scuticociliates in mesopelagic vs. euphotic waters might be related to the reprocessing of organic matter (e.g., the sinking flux of particulates and downward advection of dissolved organic matter) as they could actively feed on particulate material and bacteria (Urrutxurtu et al., 2003; Robinson et al., 2010). In addition, some scuticociliates are opportunistic or facultative parasites and can cause severe mortalities in hosts It has previously been reported that the diversity and abundance of parasitic ciliates in the meso- and abyssal-pelagic zones are higher than those in water layers above in the western Pacific Ocean (Weisse, 2017; Zhao et al., 2017). Similarly, molecular fragments of various parasitic protist groups were reported to contribute significant fractions of the total protist community in HTS datasets (Edgcomb, 2016). These findings suggest that parasitoid, parasitic and commensal protists, including oligohymenophoreans, are an important component of mesopelagic food chains.

The morphological dataset revealed a weak seasonal variation in ciliate communities (Table 2) whereas the molecular datasets (year 2013, *N* = 19;year 2014, *N* = 22; years 2013 and 2014, *N* = 41) all showed an insignificant difference in community composition between spring and summer (Table 2 and Supplementary Table S6). Looking further at community variation between spring and summer in each ecological layer using both morphological and molecular (2013 and 2014) datasets, however, revealed that significant seasonal variation only occurred at the surface layer, although the *p*-value of the former was much lower than that of the latter (Table 2 and Supplementary Table S6). Furthermore, neither the euphotic nor the mesopelagic layers showed significant difference in community composition between spring and summer (Table 2 and Supplementary Table S6). This suggests that the seasonal pattern of ciliate community was mainly the result of community variation in the surface layer. Variability in the surface ciliate community structure during different seasons is not surprising, and similar findings have been reported for surface communities of microbial eukaryotes in the north Pacific Ocean (Countway et al., 2010; Kim et al., 2014). Such variability is thought to be due to exposure to climatic fluctuations and the inherent patchiness of surface waters. At the USC Microbial Observatory (San Pedro Channel, North Pacific Ocean), a 10-year study by Kim et al. (2014) also found that seasonality of microeukaryote communities was not detected in deep (500 m) waters. By contrast, weekly sampling over a 6-month (January to June 2017) period at a deep-water (250 m) mesopelagic coastal site in the Mediterranean Sea revealed seasonal variation in tintinnid ciliate communities (Dolan et al., 2019). This variation was attributed to seasonal fluctuations in depth of the mixed layer which can extend to >200 m with deep water (>500 m) formation during winter (January–March) (Dolan et al., 2019). For the transect in the present study, the mixed layer also varied with season but within a much narrower range, i.e., by approximately 11–13 m for sites C1 and C3, 20 m for sites C5 and C7, 30 m for site C9 in spring, 6 m for site C1, 7–15 m for sites C3, C5, and C7, and 20 m for site C9 in summer. Also, compared with the surface layer, horizontal advection is much weaker in the mesopelagic zone. Therefore, the insignificant temporal variation in mesopelagic ciliates might be due to temporally stable environmental conditions at this depth.

Partial Mantel test suggested that depth, geographic distance, and environmental factors played key roles in shaping ciliate community composition, with depth being the most critical (Table 3). Although a high proportion of community variation was depth-related, this might be due to depth being a proxy for various physiochemical factors in oceanic waters. For example, with increasing depth, oceanic waters are prone to become more dense with lower temperature, higher salinity, higher concentrations of dissolved organic matter and lower concentrations of oxygen, each of which is likely to influence microbial trophic function and community structure (Jiao et al., 2013; Pernice et al., 2015; Edgcomb, 2016).

Understanding the ecological processes that shape community assembly is of central importance in the field of community biology. Most previous studies, however, have focused on either prokaryotes (Burke et al., 2011) or plants and animals (Kraft and Ackerly, 2014). The processes that shape community assembly of microeukaryotes in general (Logares et al., 2018), and ciliates in particular (Dolan et al., 2007), remain poorly understood. The employment of phylogenetic null model analysis makes determination and quantification of ecological processes possible (Stegen et al., 2013). This approach has limitations, e.g., the framework is unable to differentiate biological interaction, such as competition and trophic interactions, from selection and is subjective to methodological artifacts such as PCR-bias and DNA sequencing errors (Stegen et al., 2013). Nevertheless, it has been accepted as an effective means of analyzing community assembly of microbes in a variety of environments (Bahram et al., 2015; Dini-Andreote et al., 2015; Logares et al., 2018). This study is the first to use this framework to address the assembly mechanisms of ciliate communities. Dispersal limitation was found to be the most important ecological process shaping ciliate communities, followed by heterogeneous selection and ecological drift (Figure 3). The many physical and biogeochemical features of water across depth strata represent a highly heterogeneous set of environments that generates different vertical ecological layers and a strong response by the ciliate community to environmental selection and dispersal limitation. In surface waters, heterogeneous selection contributed a much higher proportion of the community variation than did dispersal limitation (Figure 3). This contrasts with the situation in the euphotic and mesopelagic zones, indicating that assembly mechanisms in surface waters differs from those deeper in the water column. Unlike the euphotic and mesopelagic zones, the surface is generally characterized by higher fluctuations of salinity and temperature and stronger mixing by wind leading to higher environmental filtering and lower dispersal limitations (Wu et al., 2017). On the other hand, wind-induced mixing would generate a relatively homogeneous environment (Wu et al., 2017). This is likely the reason why homogeneous dispersal was observed only in surface waters, albeit with a relatively small contribution (Figure 3). Compared with the surface layer, horizontal mixing is much weaker in deeper waters, therefore the impact of dispersal limitation is greater in the euphotic and mesopelagic zones. The transitional layer between the base of euphotic zone and the top of mesopelagic zone showed that environmental selection was primarily responsible for determining ciliate community structure. This layer represents a depth of major transitions of several environmental factors. Therefore, a stronger response of the ciliate community to selection was found in transitional layer than in other water layers.

### Mesopelagic Ciliates Across Oceanic Basins

Only a limited number of studies on spatio-temporal distributions of ciliates down the water column have been performed, especially in the mesopelagic zone (Wickham et al., 2011; Hu et al., 2016). Therefore, it is difficult to carry out regional comparisons of vertical distribution of mesopelagic ciliate diversity. By downloading 18S rRNA V4 region sequences derived from two pilot studies carried out in the western Pacific Ocean (Zhao et al., 2017) and eastern North Pacific (Hu et al., 2016) and analyzing these along with the present data from the SCS, we found that ciliate diversity in the mesopelagic zone was comparable to that of surface water in each of the three ocean basins regardless of the type of dataset analyzed, i.e., rRNA gene, rRNA gene transcript or morphology (Figure 5). This suggests that the high ciliate diversity in the mesopelagic zone is a general phenomenon. Mesopelagic communities showed a large variability across oceanic basins, as seen by the distribution of samples in the PCoA (Figure 6D) and statistical results of ANOSIM (Supplementary Table S9). Mesopelagic ciliate communities showed a clear distance-decay pattern (Supplementary Figure S7) in that the similarity of community composition decreased with geographic distance increased. This phenomenon could explain why community similarity between the two geographically close basins, i.e., northern SCS and western Pacific Ocean, was higher than those of other pairwise comparisons, i.e., northern SCS and eastern North Pacific Ocean, western Pacific and eastern North Pacific Oceans (Figure 6D and Supplementary Table S9). Similar results were reported for microeukaryotes in abyssal waters across world’s oceans, the similarity of environmental conditions shaping the microbial communities in nearby sites being cited as the most plausible cause (Pernice et al., 2015). Using the null modeling approach, it was found that heterogeneous selection was the primary factor determining mesopelagic ciliate community assembly across oceanic basins (Figure 6B). Therefore, the large proportion of unique OTUs of each basin (Figure 6C), and distinct community composition across basins (Figure 6D and Supplementary Table S9), could be mainly the result of environmental selection. This is consistent with the findings for microeukaryotes in surface (Massana and Logares, 2013) and deep waters (Pernice et al., 2015). It should be noted, however, that the distribution pattern seen in Figure 6D could also be an artifact that reflects the differences in procedures for dealing with samples between the present and the previous studies (Hu et al., 2016; Zhao et al., 2017). Study-specific factors, e.g., amount of water sampled for nucleic acid extraction, extraction and PCR protocols, and sequencing depth, could strongly influence the results. Therefore, standard operating procedures of sampling and data processing should be established and taken into consideration in future studies. Collectively, these findings suggest that mesopelagic ciliates across oceanic basins exhibit high diversity, and their biogeography could be mainly shaped by environmental selection. Future studies on a wider range of protist groups over broader temporal scales and across other ocean basins, water masses or biogeochemical provinces are needed to determine whether this pattern of diversity is widely applicable.

### Community Composition Revealed by Molecular- and Morphology-Based Methods

Previous studies indicated that HTS can detect species present in low abundance in a sample and therefore holds the potential to retrieve components of diversity not evident using traditional morphological methods (Sogin et al., 2006). In the present study, HTS revealed more diverse assemblages of ciliates than microscopic examination of specimens prepared by QPS leading to the result that the community composition disclosed by the two approaches did not match perfectly. For example, Colpodea and Phyllopharyngea contributed ca. 13.1 and 23.5% of the ciliate community in molecular datasets, respectively, but were undetected by QPS (Supplementary Figure S8a). This might be due to the differences inherent to the molecular/morphology-based methods, e.g., cell loss during sample processing and preservation (Pfister et al., 1999; Sonntag et al., 2000). Also, variation in rDNA copy number among different lineages of ciliates could confuse the comparison between morphological and molecular methods (Wang et al., 2017). In the present study, rRNA-based amplification was applied in order to mitigate the impact of this, nevertheless variations in copy number can still influence community composition based on rRNA datasets. Similarly, in a survey on ciliates from the surface to the abyssopelagic zone in the Western Pacific Ocean, Zhao et al. (2017) found Phyllopharyngea to be much more diverse and abundant in the community derived from cDNA than DNA sequencing. In addition, activity, growth rate, cell size and biomass could also contribute to varying amounts of rRNA in ciliates (Fu and Gong, 2017) and thus strongly influence the community composition revealed by the two approaches.

Overall, both methods showed the major contributors of ciliate community at class level, indicating community composition at high taxonomic level revealed by HTS and QPS are comparable (Supplementary Figure S8a). Congruence generally decreased, however, when moving down the taxonomic hierarchy from class to genus (Supplementary Figures S8a–d). Among 79 morphospecies, 78 were assignable to class, 64 to order, 64 to family and 59 to genus. For the molecular dataset, 571 of 575 OTUs were assignable to class, 368 to order, 349 to family and 227 to genus. Therefore, it is difficult to compare the differences in community composition at lower taxonomic levels revealed by the two approaches. For instance, if an OTU is lacking an affiliation to an order, then it is impossible to determine its identity at any rank below class. In the present study, approximately 19% of morphospecies and 36% of OTUs could not be assigned to any known order. Comparison of composition at lower taxonomic levels as revealed by the two approaches therefore becomes inappropriate and inaccurate under these circumstances. This also corresponds with the findings of previous studies where both methods have been applied, i.e., on tintinnid ciliates from coastal waters (Bachy et al., 2013; Santoferrara et al., 2014) or planktonic ciliates from mountain lakes (Stoeck et al., 2014; Pitsch et al., 2019), which have also reported a gap between molecular and morphological methods for revealing community composition at lower taxonomic ranks.

Unassigned/unidentified taxa generally occupy higher proportions in molecular datasets than in morphological datasets, suggesting that the reference databases for assigning environmental sequences to different taxonomic ranks remain far from being sufficiently well-represented. In this study, Oligotrichia and Choreotrichia were the top two abundant assemblages in the morphological dataset, accounting for 31.33 and 28.35% of total species abundance, respectively. For species of Oligotrichia, only four out of 22 species have a V4 18S rRNA sequence in the PR2 database (version 4.7.2). Likewise, none of the nine aloricate choreotrichians and only seven out of 19 loricate choreotrichians could be related to a named sequence in the database. The lack of sufficient sequence information for a given morphospecies might also contribute to the discrepancy between morphological and molecular datasets. Furthermore, errors in the reference database could also confuse the interpretation, such as: (i) taxonomic classification is not always consistent at each rank, e.g., name of subclass occurred at the place where order name should be given; (ii) the classification used is inconsistent as taxonomic and molecular classifications are sometimes incongruent; (iii) sequences annotated with a species name sometimes have ambiguous names at higher taxonomic ranks; (iv) multiple sequences may bear the same species name, e.g., 15 different sequences are registered in the database under the species name *Peritromus kahli*. Therefore, the discrepancy between ciliate community composition revealed by HTS and QPS seen in this study is consistent with previous findings for other assemblages of microbial eukaryotes (Egge et al., 2013). This suggests that direct comparisons in community composition revealed by the two methods at low taxonomic ranks are not applicable.

Despite the inherent limitation of the comparison between morphological and molecular methods, the distribution patterns of the ciliate communities disclosed by the two methods generally agree with each other (Figure 1C, Supplementary Figure S3c, Table 2, and Supplementary Table S6). Both approaches revealed a vertical distribution pattern, and a seasonal variation for surface ciliate communities, confirming the existence of a spatiotemporal distribution pattern the studied area. Similar findings were also reported for tintinnid ciliates across a coast-to-ocean transect off the coast of Rhode Island (United States) (Santoferrara et al., 2016). Employing morphological and molecular approaches, there was general agreement in the distribution patterns recovered and in the correlations with environmental parameters (Santoferrara et al., 2016). In the present study, however, the molecular method showed a nearshore-offshore distribution whereas this was not recovered by the morphological method (Table 2 and Supplementary Table S6). This could be due to the differences inherent in the two approaches discussed earlier. For example, members of abundant and rare species can be detected by HTS but this is subject to variation of rRNA gene copy number between individuals/species (Wang et al., 2017). QPS is exempt from the issue of rRNA gene copy numbers but has low power in detecting rare species (Bachy et al., 2013; Santoferrara et al., 2016). Therefore, it is recommended that an integrated approach, combining morphological and molecular methods, should be employed to study the diversity and distribution of ciliates in order to mitigate the limitations of using one method alone.

In summary, mesopelagic ciliates exhibited comparable alpha diversity to that of surface communities in the northern SCS and this distribution pattern was consistent temporally and across two other oceanic basins. Mesopelagic ciliates harbored distinct community structure with insignificant differences occurring temporally. Additionally, dispersal limitation was found to override heterogeneous selection in determining mesopelagic ciliate community assembly. Due to the number of mesopelagic samples collected being limited to two sites, the aim of identifying the spatially dynamic pattern of mesopelagic ciliates in the SCS could not be met. In future studies, spatial sampling on a wider scale should be conducted to confirm the patterns observed here. An integrative approach combining multiple sources of information, e.g., morphology, molecular and ecology, is needed in order to characterize the community dynamics of mesopelagic ciliates.

## Data Availability Statement

The datasets generated for this study can be found in the NCBI Sequence Read Archive (Accession Code SRP182690).

## Author Contributions

PS conceived and designed the study. LH conducted the experiments. LW, WX, and JK collected the samples. PS, LH, and YW analyzed the data. All authors wrote the manuscript.

## Conflict of Interest

The authors declare that the research was conducted in the absence of any commercial or financial relationships that could be construed as a potential conflict of interest.

## Footnotes

1 http://vegan.r-forge.r-project.org/
