# Supplementary material for: Integrated Space-Time Dataset Reveals High Diversity and Distinct Community Structure of Ciliates in Mesopelagic Waters of the Northern South China Sea
Source: Front Microbiol. 2019 Sep 24;10:2178. doi: 10.3389/fmicb.2019.02178 (PMC6768975; doi:10.3389/fmicb.2019.02178)
Supplement: Supplementary file 1 [file Data_Sheet_1.PDF]

Supplementary Figure S1 | Principal component analysis (PCA) showing environmental gradients from coastal to oceanic and surface to deep waters. Inset showing map of the northern South China Sea highlighting the transect of the stations sampled.

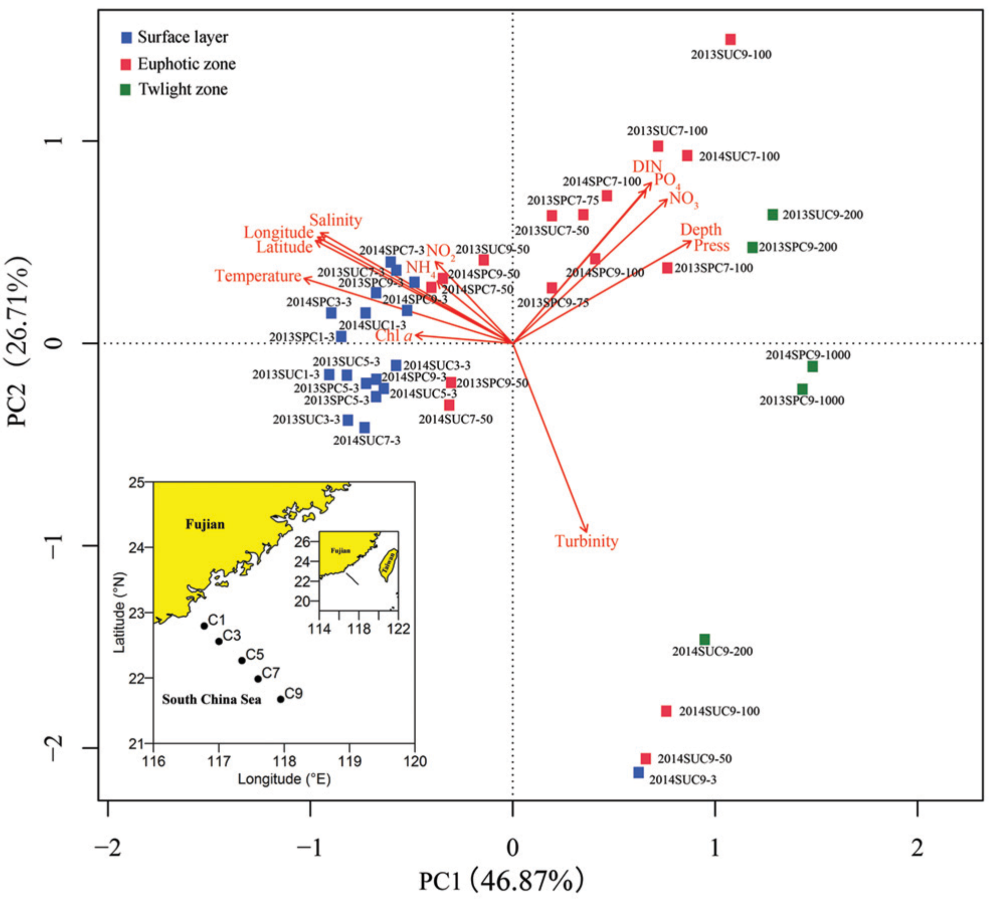

Supplementary Figure S2 | Ciliate OTU richness (upper), Shannon (middle) and effective number of species (ExpH', lower) indices in vertical dimensions for molecular (a) and morphological (b) data of the year 2014.

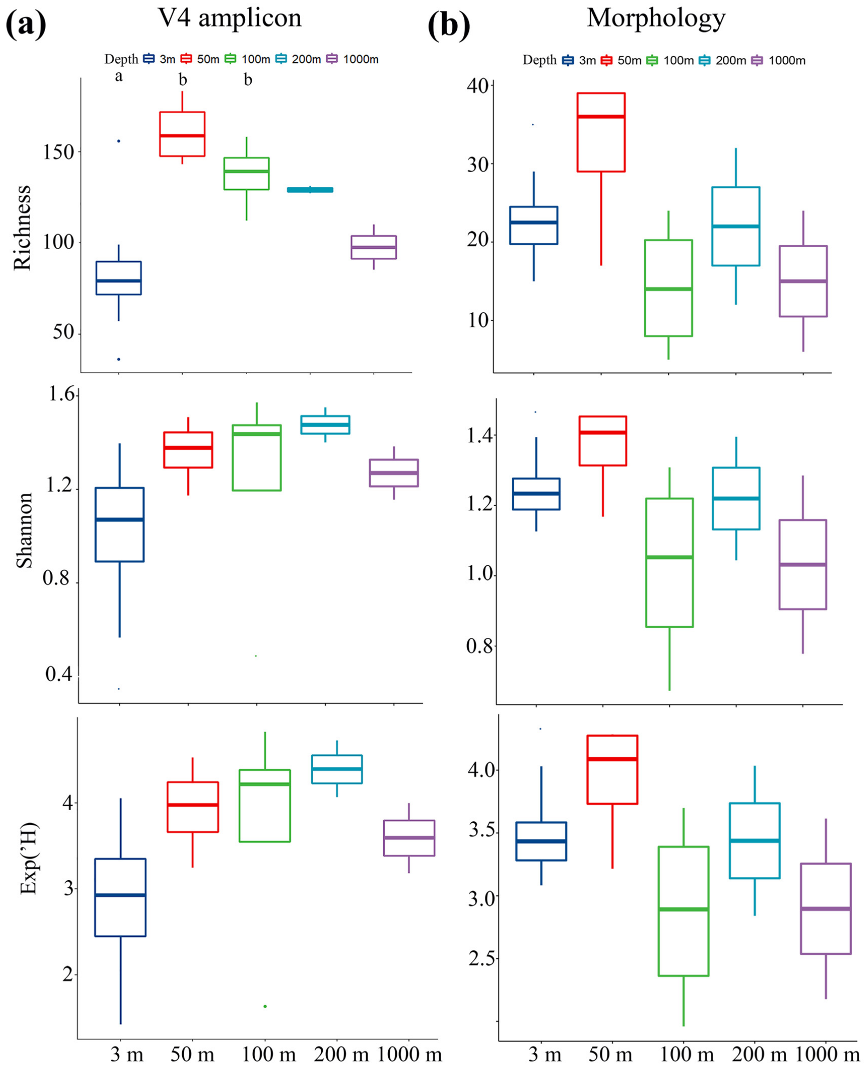

Supplementary Figure S3 | (a) OTU richness in vertical (upper, left), horizontal (upper, right), seasonal (lower, left) and annual (lower, right) dimensions for the pooled rRNA data as demonstrated by boxplots with median and 95% confidence intervals displayed. Significant differences for pairwise comparisons are also shown. (b) OTU richness (upper), effective number of species (ExpH', middle) and phylogenetic diversity (PD, lower)) indices in vertical distribution for the pooled data of the years 2013 and 2014. (c) Non-metric multidimensional scaling (nMDS) plot of all pooled samples for the years 2013 and 2014 on the basis of Bray-Curtis dissimilarity.

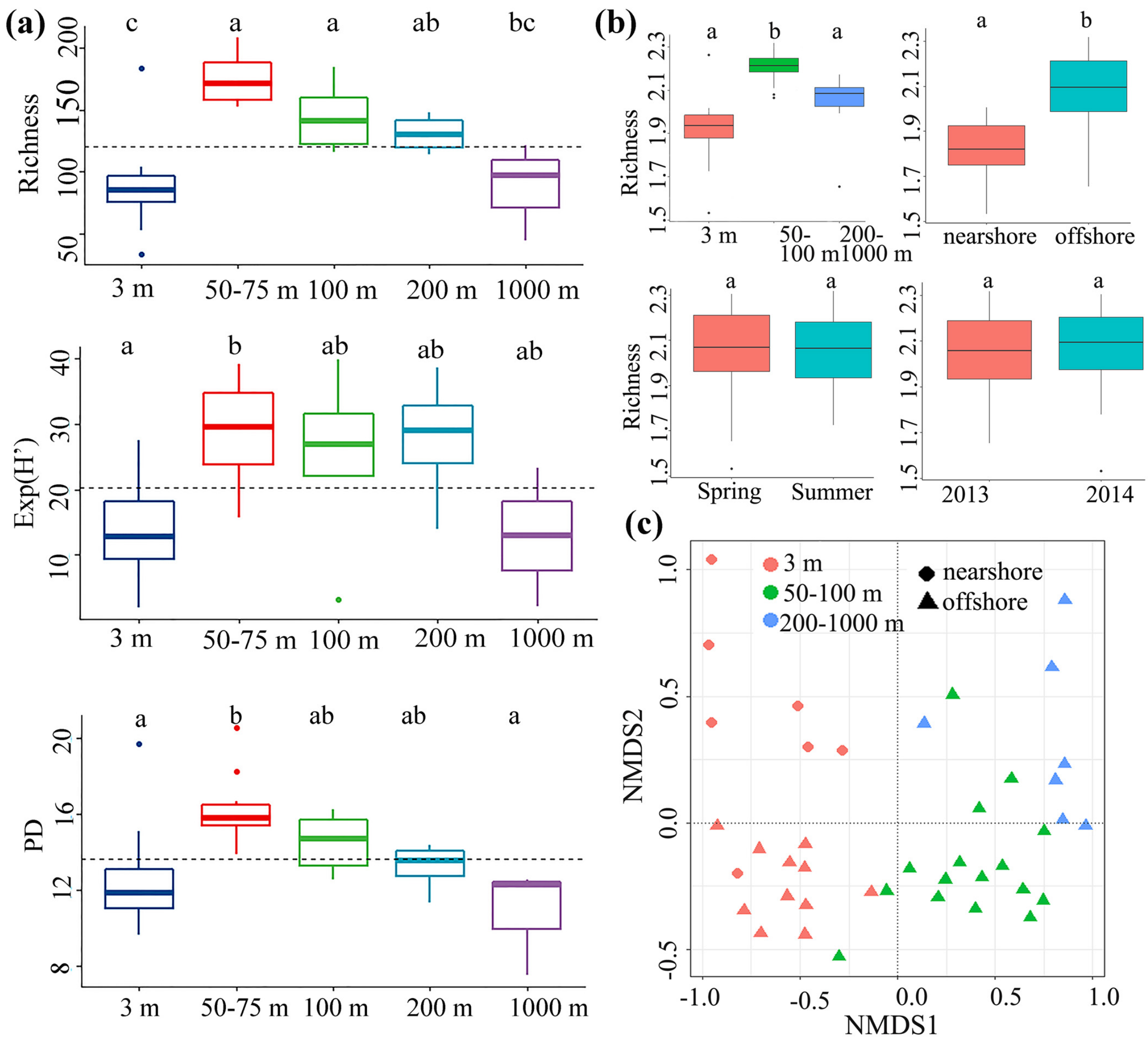

Fig. S4 | Relative proportion of the identified classes of the ciliate communities in each ecological layer revealed by morphological (a)and molecular (b, c) approaches, respectively. 2014 QPS: dataset of year 2014 based on QPS method; 2014 HTS: dataset of year 2014 based on HTS method; 2013-2014 HTS: dataset of years 2013 and 2014 based on HTS method.

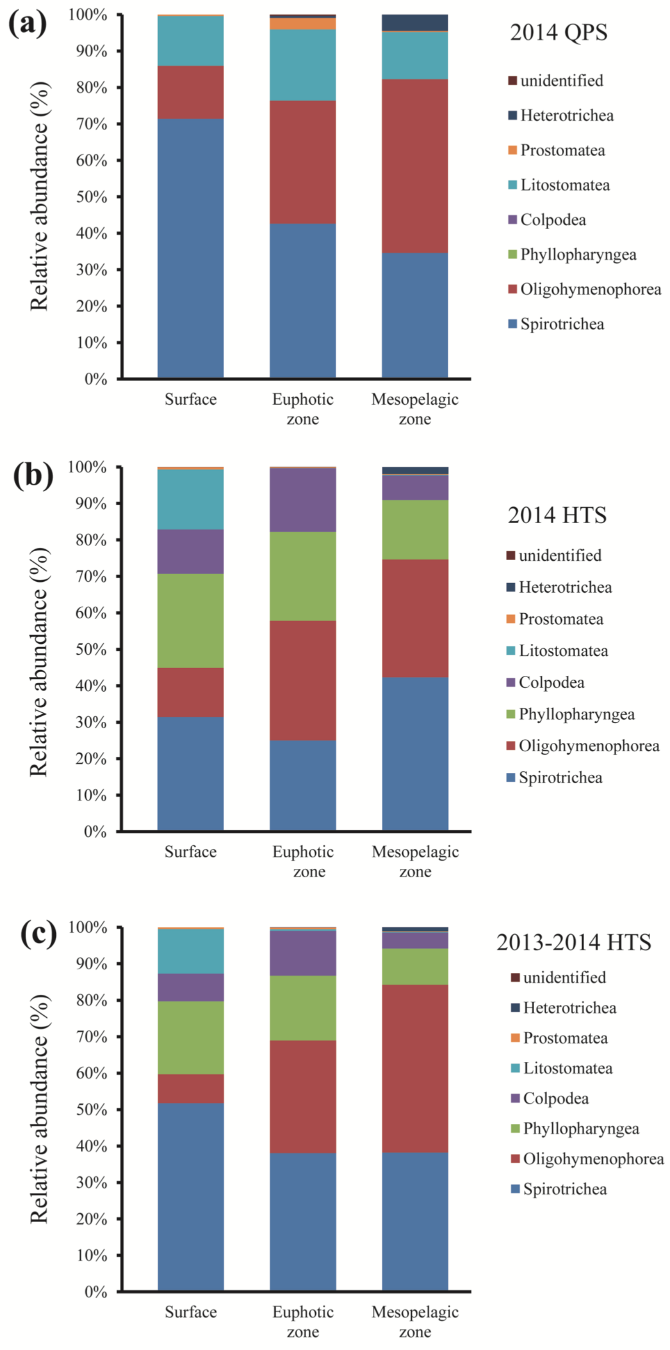

Fig. S5 | Correlation of community dissimilarity with depth (a), geographical distance (b) and environmental distance (c).

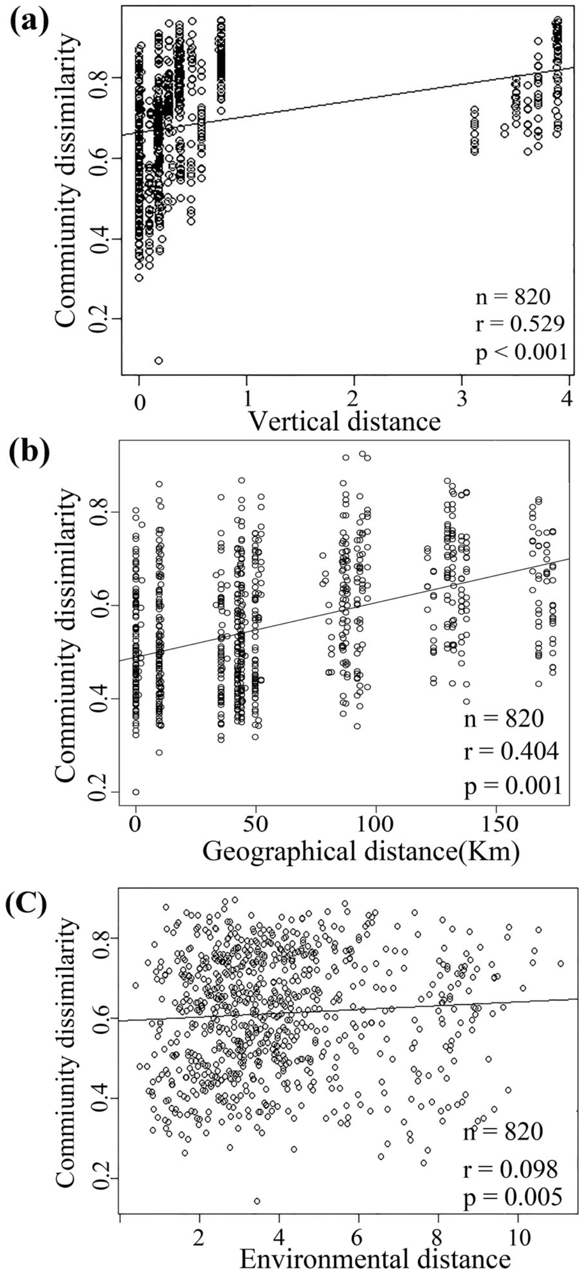

Fig. S6 | Ciliate abundance along the transect for the year 2014 as revealed by QPS approach.

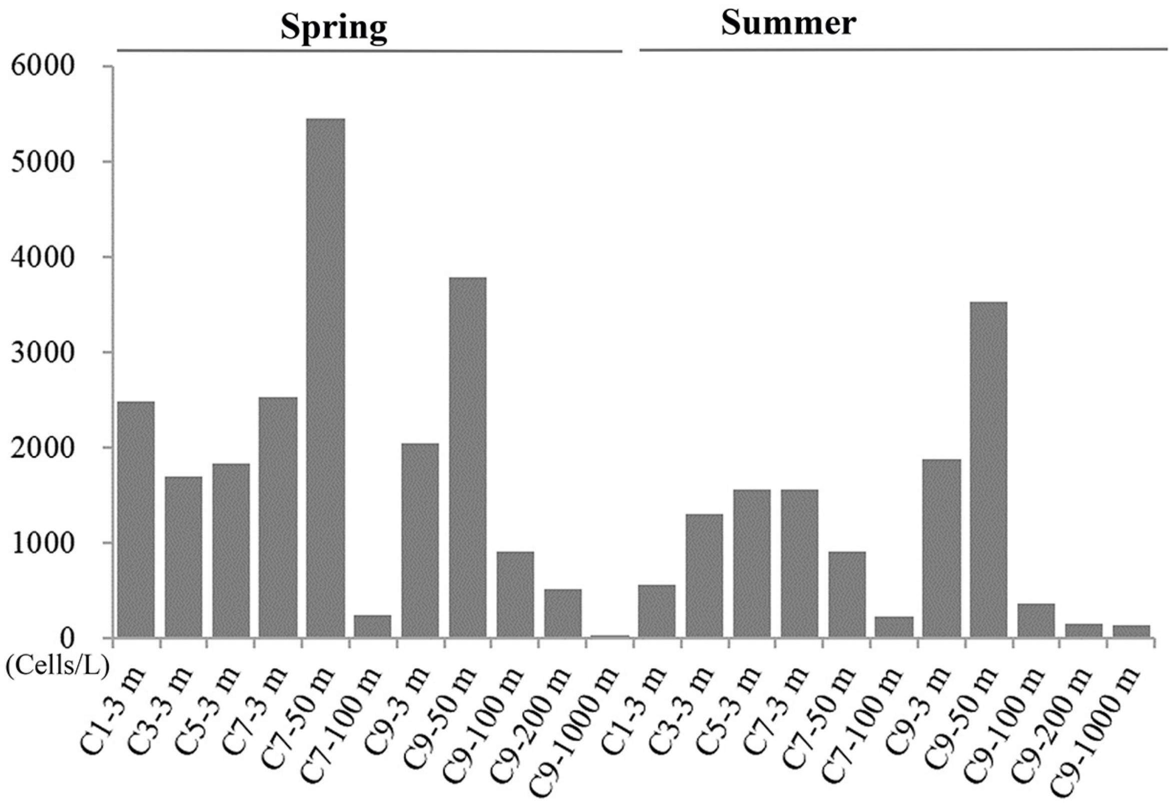

Figure S7 | Relationship between geographic distances and community similarities of mesopelagic ciliates of three oceanic basins.

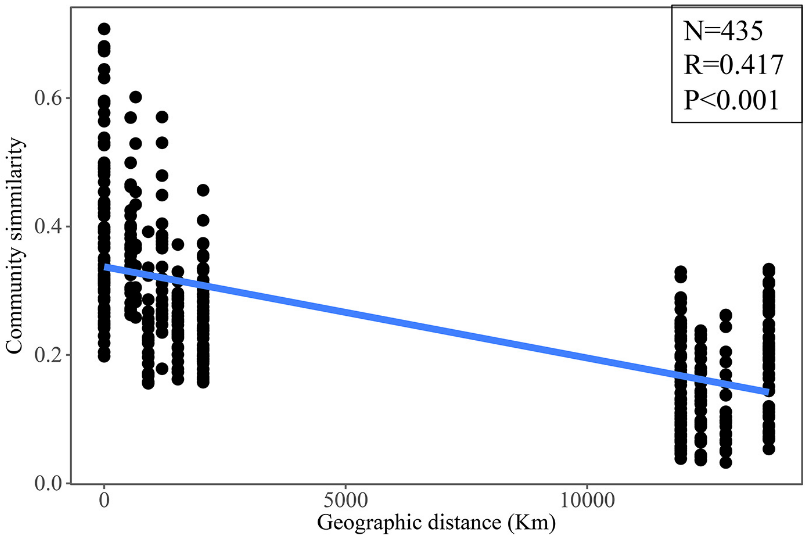

Figure S8 | Molecular and morphology examination of assemblage compositions of ciliate community of the year 2014 at class, order, family and genus levels.

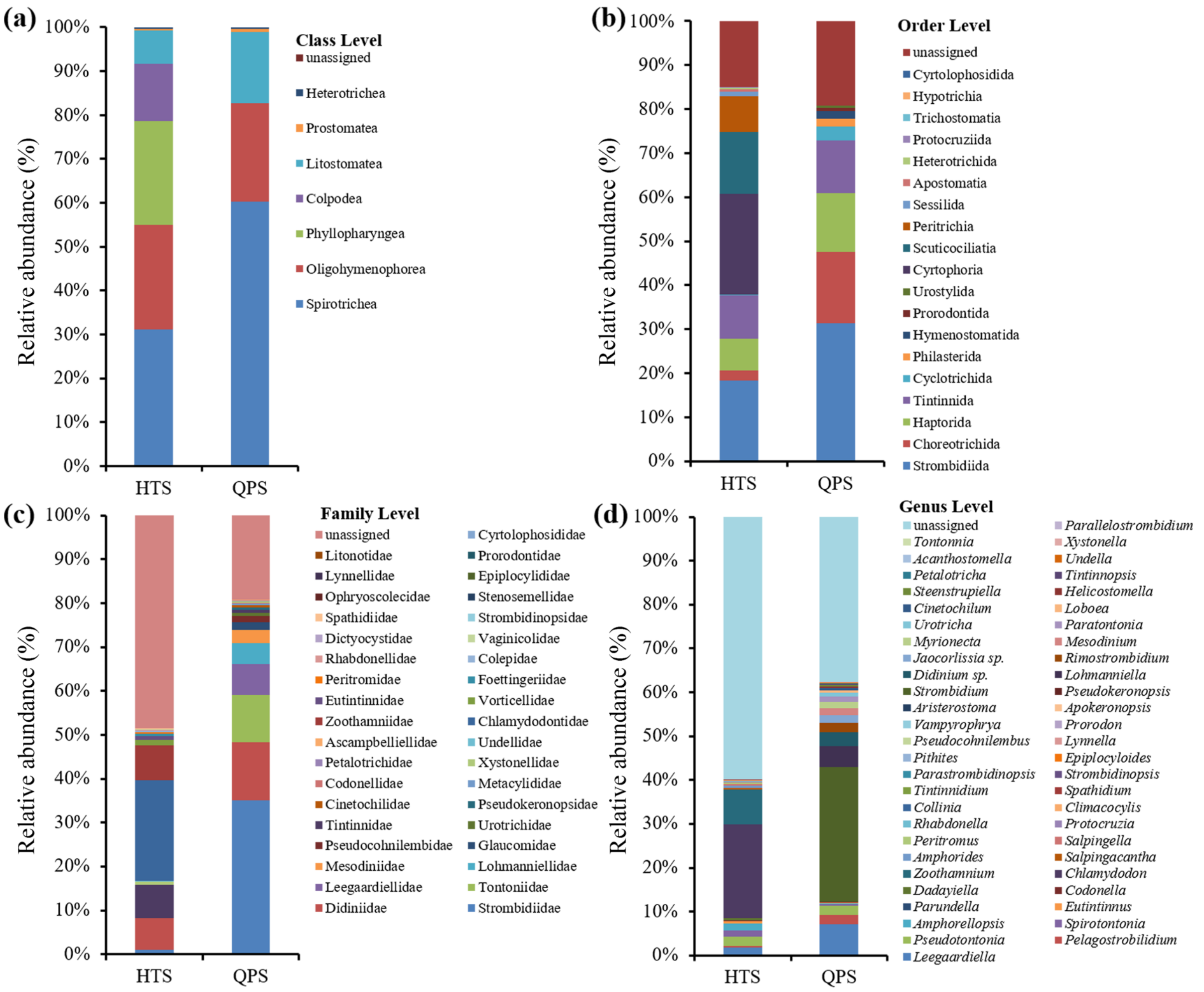

Table S1. Information of the sampling sites along the transect C.

	Sampling site	Longitude	Latitude	Sampling Depth (m)	Maximum Water Depth (m)	Sampling Year	Sampling Season
1	C1	116.78°E	22.80°N	3	37	2013	Summer
2						2014	Spring
3						2014	Summer
4	C3	117.01°E	22.56°N	3	37	2013	Spring
5						2013	Summer
6						2014	Spring
7						2014	Summer
8	C5	117.31°E	22.27°N	3	59	2013	Spring
9						2013	Summer
10						2014	Spring
11						2014	Summer
12	C7	117.60°E	21.98°N	75	126	2013	Spring
13				100		2013	Spring
14				3		2013	Summer
15				50		2013	Summer
16				75		2013	Summer
17				3		2014	Spring
18				50		2014	Spring
19				100		2014	Spring
20				3		2014	Summer
21				50		2014	Summer
22				103		2014	Summer
23	C9	117.95°E	21.67°N	3	1280	2013	Spring
24				50		2013	Spring
25				75		2013	Spring
26				200		2013	Spring
27				1000		2013	Summer
28				3		2013	Summer
29				50		2013	Summer
30				100		2013	Summer
31				200		2013	Spring
32				3		2014	Spring
33				50		2014	Spring
34				100		2014	Spring
35				200		2014	Spring
36				1000		2014	Summer
37				3		2014	Summer
38				50		2014	Summer
39				100		2014	Summer

40	200	2014	Summer
41	1000	2014	Summer

Supplementary Table S2 | Information of samples collected from eastern North Pacific and western Pacific Ocean. RGT: rRNA gene transcript; RG: rRNA gene.

Eastern North Pacific				Western Pacific Ocean			
Sampling names	Location	Depth	Nucleic acid	Sampling names	Location	Depth	Nucleic acid
SRR3180319	Catalina	5 m	RGT	SRR5675497	DY12	DCM	RG
SRR3180321	SPOT	5 m	RGT	SRR5675498	DY12	200 m	RG
SRR3180323	Catalina	5 m	RGT	SRR5675499	DY1	2000 m	RG
SRR3180325	Port of LA	5 m	RGT	SRR5675500	DY7	2 m	RG
SRR3180327	SPOT	150 m	RGT	SRR5675501	DY7	DCM	RG
SRR3180329	SPOT	890 m	RGT	SRR5675502	DY7	200 m	RG
SRR3180331	Port of LA	5 m	RGT	SRR5675503	DY7	1000 m	RG
SRR3180332	SPOT	DC m	RGT	SRR5675504	DY7	2000 m	RG
SRR3180338	SPOT	150 m	RGT	SRR5675505	DY7	4981 m	RG
SRR3180345	SPOT	5 m	RGT	SRR5675506	DY12	2 m	RG
SRR3180343	SPOT	DC m	RGT	SRR5675507	DY1	200 m	RG
SRR3180340	SPOT	890 m	RGT	SRR5675508	DY1	1000 m	RG
SRR3180351	Catalina	5 m	RGT	SRR5675509	DY10	2 m	RG
SRR3180349	Port of LA	5 m	RGT	SRR5675510	DY11	2 m	RG
SRR3180347	Catalina	5 m	RGT	SRR5675511	DY1	2 m	RG
SRR3180356	SPOT	150 m	RGT	SRR5675512	DY1	DCM	RG
SRR3180354	Port of LA	5 m	RGT	SRR5675513	DY3	2 m	RG
SRR3180353	SPOT	150 m	RGT	SRR5675514	DY6	2 m	RG
SRR3180362	SPOT	5 m	RGT	SRR5675515	DY8	2 m	RG
SRR3180360	SPOT	DC m	RGT	SRR5675516	DY9	2 m	RG
SRR3180358	SPOT	890 m	RGT	SRR5675517	DY12	200 m	RGT
SRR3180367	SPOT	DC m	RGT	SRR5675518	DY12	DCM	RGT
SRR3180365	SPOT	890 m	RGT	SRR5675519	DY12	2000 m	RGT
SRR3180334	SPOT	5 m	RGT	SRR5675520	DY12	1000 m	RGT
SRR3180335	SPOT	5 m	RGT	SRR5675521	DY12	2000 m	RG

SRR5675522	DY12	1000 m	RG
SRR5675523	DY12	2 m	RGT
SRR5675524	DY12	4765 m	RG
SRR5675525	DY12	4765 m	RGT

Supplementary Table S3 | Spearman's rank correlation between variation of each environmental parameters (Euclidean distance) and geographic distance/depth of each sampling site.

	Geographic distance		Depth		
	r	P	r	P	N
Pressure	**0.223**	**<0.001**	**0.997**	**<0.001**	41
Temperature	-0.083	0.883	**0.571**	**<0.001**	41
Salinity	**0.375**	**<0.001**	**0.324**	**<0.001**	41
Turbidity	-0.009	0.486	0.061	0.141	41
DO	-0.018	0.516	**0.335**	**<0.001**	31
PH	-0.053	0.666	**0.252**	**<0.001**	31
Bacteria	0.148	0.091	**0.668**	**<0.001**	22
Chl *a*	**0.211**	**0.005**	0.08	0.061	41
PO_4-P	-0.049	0.713	**0.582**	**<0.001**	41
DIN	-0.091	0.899	**0.602**	**<0.001**	41
NO_2-N	-0.022	0.581	-0.072	0.98	41
NO_3-N	-0.096	0.919	**0.612**	**<0.001**	41
NH_4-N	-0.016	0.519	0.06	0.101	41

Table S4 | Comparison of operational taxonomic unit (OTU) richness in vertical, horizontal and seasonal dimensions revealed by molecular and morphological approaches for the year 2014.

Pairs	Methods	p (V4 amplicon)	p (Morphology)	N
Horizontal dimension				
Nearshore vs. offshore	t.test	**0.002**	0.708	nearshore=4, offshore=18
Vertical dimension				
Surface vs. euphotic zone	ANOVA	**<0.001**	1.000	surface=10, euphotic zone=8
Surface vs. mesopelagic zone	ANOVA	0.158	0.719	surface=10, mesopelagic zone=4
Euphotic zone vs. mesopelagic zone	ANOVA	0.099	0.733	euphotic zone=8, mesopelagic zone=4
Temporal comparison				
Spring vs. summer	t.test	0.783	0.467	spring=11, summer=11

Supplementary Table S5 | SIMPER analysis showed class contributions to the vertical community rearrangements based on molecular and morphological dataset for the year 2014.

Class	V4 amplicon	Morphology
Spirotrichea	29.05%	62.03%
Oligohymenophorea	27.57%	21.62%
Phyllopharyngea	22.05%	-
Colpodea	12.28%	-
Litostomatea	8.01%	14.63%
Heterotrichea	0.57%	0.44%
Prostomatea	0.46%	1.20%
others	0.01%	0.08%

Supplementary Table S6 | ANOSIM statistical tests of the groupings of molecular-based ciliate communities for the years 2013 and 2014 according to depth, region, season and year. Community turnover is based on the Bray-Curtis distance.

	2013_2014_V4		N
	R	P	
Depth	0.694	**<0.001**	N=44
3 m vs. 50-100 m	0.699	**<0.001**	N(3 m)=18, N(50-100 m)=16
3 m vs. 200-1000 m	0.923	**<0.001**	N(3 m)=18, N(200-1000 m)=7
50-100 m vs. 200-1000 m	0.547	**<0.001**	N(50-100 m)=16, N(200-1000 m)=7
Nearshore vs. offshore	0.432	**<0.001**	N(nearshore)=7, N(offshore)=34
Season (spring vs. summer)	0.039	0.113	N(spring)=20, N(summer)=21
Year (2013 vs. 2014)	0.058	0.071	N(2013)=19, N(2014)=22
Spring vs. summer (3 m)	0.128	**0.045**	N(spring)=8, N(summer)=10
Spring vs. summer (50-100 m)	0.127	**0.039**	N(spring)=8, N(summer)=8
2013 vs. 2014 (3 m)	0.181	**0.026**	N(2013)=8, N(2014)=10
2013 vs. 2014 (50-100 m)	0.121	0.053	N(2013)=8, N(2014)=8
2013 vs. 2014 (200-1000 m)	0.074	0.367	N(2013)=3, N(2014)=4

Supplementary Table S7 | Taxonomy and ecological distribution of species revealed by QPS approach.

Species name	Abundance(cells/L)	Sites	Depth(m)
Acanthostomella obtusa	39	C7;C9	50;100
Amphorellopsis acuta	75	C3;C9	3
Cinetochilum sp.	174	C7;C9	50
Codonella nationalis	33	C9	50
Dadayiella ganymedes	7	C7	100
Didiniidae_X sp.	3404	C1;C3;C5;C7;C9	3;50;100;200;1000
Didinium sp1	910	C1;C3;C7;C9	3;50;100;200;
Didinium sp2	166	C7;C9	50;100
Eutintinnus fraknoi	52	C9	3;200;1000
Haptoria_X_X_X sp.	2	C9	1000
Helicostomella subulata	120	C7;C9	3;50
Heterotrichea_X_X_X_X sp.	90	C7;C9	50;200;1000
Hymenostomatia_X_X_X sp.	314	C1;C3;C5;C9	3;200;1000
Jaocorlissia sp.	585	C1;C3;C5;C7;C9	3;50;200;1000
Leegaardiella cf. sol	341	C5;C7;C9	3;100;200;1000
Leegaardiella ovalis	1100	C1;C3;C5;C7;C9	3;50;100;200;1000
Leegaardiella sol	221	C7;C9	50;100
Leegaardiella sp.	717	C1;C3;C5;C7;C9	3;50;100
Loboea strobila	197	C1;C7;C9	3;200
Lohmanniella oviformis	875	C1;C3;C5;C7;C9	3;50;200
Lohmanniella sp1	264	C7;C9	3;50;100;1000
Lohmanniella sp2	489	C1;C3;C7;C9	3;50;100
Mesodinium sp.	556	C1;C3;C5;C7;C9	3;50;100
Myrionecta rubra	475	C1;C3;C5;C7;C9	3;50;200
Parallelostrombidium kahli	10	C3	3
Paratontonia gracillima	409	C7;C9	3;50;100;1000
Parundella sp.	33	C9	50
Pelagostrobilidium sp.	769	C1;C3;C5;C7;C9	3;50;100;1000
Petalotricha sp.	57	C7;C9	50;100;200
Pseudocohnilembidae_X sp.	437	C3;C5;C7;C9	3;50;100;1000
Pseudokeronopsidae_X sp.	179	C7;C9	50;100
Pseudotontonia cornuta	704	C1;C3;C5;C7;C9	3;50;200
Rimostrombidium multinucleatum	314	C3;C7;C9	3;50;200
Rimostrombidium undinum	404	C5;C7;C9	3;50;200
Scuticociliatia_X_X_X sp.	6065	C1;C3;C5;C7;C9	3;50;100;200;1000
Spirotontonia grandis	44	C7	3
Spirotontonia sp.	10	C3	3
Spirotontonia turbinata	33	C3	3
Steenstrupiella intumescents	113	C1;C7	3;50
Strombidinopsis sp.	49	C3;C9	3;50;200
Strombidium bilobum	866	C3;C5;C7;C9	3;50;100;1000

strombidium capitatum	240	C1;C7;C9	3;50
Strombidium cf. inclinatum	348	C3;C5;C7;C9	3;100
Strombidium cf. wulffi	1081	C1;C3;C5;C7;C9	3;50;200
Strombidium conicum	378	C1;C3;C7;C9	3;50
Strombidium constrictum	177	C1;C3;C7;C9	3;50;200;1000
Strombidium dalum sensu	578	C3;C5;C7;C9	3;50;100;200
Strombidium emergens	545	C1;C3;C7;C9	3;50;100;200
Strombidium epidemum	284	C1;C5;C7;C9	3;50;100;200;1000
Strombidium pollostomum	131	C5;C7;C9	3;50
Strombidium sp1	526	C3;C5;C7;C9	3;50
Strombidium sp2	33	C9	50
Strombidium sp3	1006	C3;C5;C7;C9	3;50;100;200;1000
Strombidium sp4	44	C9	50
Strombidium sp5	2169	C1;C3;C5;C7;C9	3;50;100;200;1000
Strombidium sp6	628	C1;C3;C7;C9	3;50;200
Strombidium sp7	1191	C1;C3;C5;C7;C9	3;50;200
Strombidium styliferum	72	C7;C9	50;100
Strombidium taylori	33	C9	50
Tintinnopsis parva	60	C1	3
Tontoniidae_X sp.	2188	C1;C3;C5;C7;C9	3;50;100;200
Tontonnia gracillima	22	C3	3
Undella sp.	44	C3	3
Undella turgida	10	C3	3
Urotricha sp.	258	C3;C7;C9	50;100;200
Xystonella sp.	33	C7	50
Unidentified	7	C7	100

Supplementary Table S8 | Comparison of OTU richness, phylogenetic diversity (PD) and Shannon indices of mesopelagic ciliates across basins using ANOVA statistical tests. Samples were collected from eastern North Pacific, western Pacific Ocean and northern South China Sea, respectively. RGT: rRNA gene transcript; RG: rRNA gene.

			Richness p	PD p	Shannon p
Western Pacific Ocean (RG)	vs.	Eastern North Pacifc (RGT)	0.262	**0.008**	0.445
Western Pacific Ocean (RGT)	vs.	Eastern North Pacifc (RGT)	0.067	**<0.001**	0.106
Northern South China Sea (RGT)	vs.	Eastern North Pacifc (RGT)	0.848	0.080	0.702
Western Pacific Ocean (RGT)	vs.	Western Pacific Ocean (RG)	0.609	0.123	0.575
Northern South China Sea (RGT)	vs.	Western Pacific Ocean (RG)	0.786	0.900	0.991
Northern South China Sea (RGT)	vs.	Western Pacific Ocean (RGT)	0.263	0.058	0.492

Supplementary Table S9 | ANOSIM statistical tests of groupings of mesopelagic ciliate communities derived from samples collected from eastern North Pacific, western Pacific Ocean and northern South China Sea according to region. Community turnover is based on the Bray-Curtis distance. RGT: rRNA gene transcript; RG: rRNA gene.

			R	p
Total			0.788	**0.001**
Northern South China Sea (RGT)	vs.	Eastern North Pacifc (RGT)	0.832	**0.001**
Northern South China Sea (RGT)	vs.	Western Pacific Ocean (GT)	0.813	**0.001**
Northern South China Sea (RGT)	vs.	Western Pacific Ocean (RGT)	0.402	**0.018**
Eastern North Pacifc (RGT)	vs.	Western Pacific Ocean (GT)	0.989	**0.001**
Eastern North Pacifc (RGT)	vs.	Western Pacific Ocean (RGT)	0.903	**0.006**
Western Pacific Ocean (GT)	vs.	Western Pacific Ocean (RGT)	0.300	**0.039**
